# Supplementary material for: 6-Polyaminosteroid Squalamine Analogues Display Antibacterial Activity against Resistant Pathogens
Source: Int J Mol Sci. 2023 May 10;24(10):8568. doi: 10.3390/ijms24108568 (PMC10218190; doi:10.3390/ijms24108568)

Supplementary Materials

Experimental section

All solvents were purified according to reported procedures, and the reagents used were commercially available. Methanol, ethyl acetate, dichloromethane was purchased from Sigma and used without further purification. Column chromatography was performed on Macherey-Nagel silica gel (70-230 mesh). ^{1}H NMR and ^{13}C NMR spectra were recorded in MeOD on a Bruker AC 300 spectrometer working at 300 MHz and 75 MHz, respectively (the usual abbreviations are used: s: singlet, d: doublet, t: triplet, q: quadruplet, m: multiplet). Tetramethylsilane was used as internal standard. All chemical shifts are given in ppm. Mass spectroscopy analyses have been performed by the Spectropole (Analytical Laboratory) of Aix-Marseille university (Marseille). The purity of the compounds was checked by analytical HPLC (C18 column, eluent CH_3CN-water-TFA (90:10:0.025, v/v/v), 0.5-1 mL/min) with PDA detector spanning from 210 nm to 310 nm. All compounds possessed purity above 95%, as determined by analytical LCMS Agilent.

General procedure for the titanium–mediated reductive amination reaction of 4f: A mixture of 6-ketocholestanol (157 mg, 0.39 mmol), titanium(IV) isopropoxide (573 µL, 2.02 mmol) and spermine (202 mg, 1 mmol) in absolute methanol (5 mL) was stirred under argon at room temperature for 12 h. Sodium borohydride (38 mg, 1 mmol) was then added at -78 ºC and the resulting mixture was stirred for an additional 2 hours. The reaction was then quenched by adding water (1 mL) and stirring was maintained at room temperature for 20 minutes. The resulting inorganic precipitate was filtered off over a pad of Celite and washed with methanol and ethylacetate. The combined organic extracts were dried over Na_2SO_4, filtered, and concentrated in vacuo to afford the expected crude amino derivative which was purified by flash chromatography affording the expected amino derivative. Purification by column chromatography (silica gel; CH_2Cl_2/ MeOH/ NH_4OH(32%), 7:3:1) afforded a pale yellow solid in 45% yield; this compound can be converted subsequently into its hydrochloride salt as white solid –^{1}H NMR (300 MHz, CD_3OD) : δ = 2.65 (m, 17H), 1.90 (dt, J = 2.88, 13.72 Hz, 3H), 1.63 (m, 19H), 1.42 (m, 5H), 1.16 (dtt, J = 4.30, 9.54, 14.82 Hz, 4H), 0.95 (m, 18H), 0.76 (s, 4H). – ^{13}C NMR (75 MHz, CD_3OD) : δ = 72.41, 60.53, 57.80, 57.52, 56.27, 50.70, 50.57, 49.68, 48.26, 43.91, 41.42, 40.75, 40.62, 40.30, 37.46, 37.22, 36.81, 36.68, 33.27, 32.26, 31.81, 30.41, 29.40,

29.18, 28.34, 28.29, 25.48, 25.07, 23.30, 23.06, 22.27, 19.39, 16.81, 12.76. $C_{37}H_{72}N_4O$; MS (ESI) m/z = 589.5 $[M+H]^+$

(3β,6β)-6-[(2-aminoethyl)amino]cholestan-3-ol 4a: Purification by column chromatography (silica gel; CH_2Cl_2/ MeOH/ NH_4OH(32%), 7:3:1) afforded a pale yellow solid in 96% yield; – ^{1}H NMR (300 MHz, MeOD): δ = 3.53 (m, 1H), 2.92 (m, 2H), 2.77 (m, 4H), 2.47 (dddd, *J* = 3.02, 5.77, 6.41, 7.32 Hz, 1H), 1.29 (m, 28H), 0.92 (dd, *J* = 1.54, 6.68 Hz, 4H), 0.84 (d, *J* = 6.70 Hz, 4H), 0.66 (m, 6H). – ^{13}C NMR (75 MHz, MeOD): δ = 71.57, 58.79, 58.61, 56.28, 56.00, 54.74, 50.95, 47.27, 42.62, 41.88, 39.93, 39.48, 39.00, 36.23, 36.14, 36.05, 35.75, 35.64, 31.54, 30.39, 27.96, 24.36, 23.79, 22.76, 22.52, 21.03, 18.63, 15.21, 12.12. $C_{29}H_{54}N_2O$; MS (ESI) m/z = 447.3 $[M+H]^+$

(3β,6β)-6-[(3-aminopropyl)amino]cholestan-3-ol 4b: Purification by column chromatography (silica gel; CH_2Cl_2/ MeOH/ NH_4OH(32%), 7:3:1) afforded a pale yellow solid in 68% yield; – ^{1}H NMR (300 MHz, MeOD): δ 3.57 (m, 1H), 2.78 (tddd, *J* = 4.03, 5.41, 13.92, 19.41 Hz, 2H), 2.58 (tt, *J* = 4.81, 6.15 Hz, 2H), 1.37 (m, 34H), 0.82 (dd, *J* = 1.54, 6.68 Hz, 3H), 0.74 (d, *J* = 6.70 Hz, 5H), 0.63 (m, 6H). – ^{13}C NMR (75 MHz, MeOD): δ = 71.54, 60.35, 56.25, 56.22, 51.86, 48.35, 46.55, 42.84, 39.80, 39.50, 37.65, 36.89, 36.12, 35.68, 34.59, 33.33, 32.97, 32.77, 31.35, 29.18, 29.01, 28.29, 24.48, 24.36, 22.46, 21.19, 18.86, 15.99, 12.16. $C_{33}H_{56}N_2O$; MS (ESI) m/z = 461.4 $[M+H]^+$

(3β,6β)-6-[(4-aminobutyl)amino]cholestan-3-ol 4c: Purification by column chromatography (silica gel; CH_2Cl_2/ MeOH/ NH_4OH(32%), 7:3:1) afforded a pale yellow solid in 73% yield; – ^{1}H NMR (300 MHz, MeOD): δ = 3.61 (m, 1H), 2.97 (tt, *J* = 4.99, 6.52 Hz, 2H), 2.81 (m, 4H), 1.32 (m, 30H), 0.92 (dd, *J* = 1.54, 6.68 Hz, 4H), 0.81 (d, *J* = 6.70 Hz, 4H), 0.66 (m, 6H) – ^{13}C NMR (75 MHz, MeOD): δ = 71.65, 59.88, 58.54, 56.29, 56.04, 54.75, 48.18, 47.29, 42.71, 42.64, 39.94, 39.50, 39.04, 36.16, 35.78, 35.65, 31.56, 31.03, 30.40, 29.67, 27.99, 25.96, 24.35, 23.81, 22.79, 22.54, 21.05, 18.65, 16.33, 14.09, 12.15. $C_{31}H_{58}N_2O$; MS (ESI) m/z = 475.8 $[M+H]^+$

(3β,6β)-6-[(5-aminopenthyl)amino]cholestan-3-ol 4d: Purification by column chromatography (silica gel; CH_2Cl_2/ MeOH/ NH_4OH(32%), 7:3:1) afforded a pale yellow solid in 90% yield; – ^{1}H NMR (300 MHz, MeOD): δ = 3.58 (m, 1H), 2.91 (m, 2H), 2.69 (tt, *J* = 5.22,

6.49 Hz, 2H), 1.91 (m, 3H), 1.79 (m, 7H), 1.45 (m, 8H), 1.20 (m, 18H), 0.93 (dd, *J* = 1.54, 6.68 Hz, 3H), 0.84 (d, *J* = 6.70 Hz, 5H), 0.62 (m, 7H).– ^{13}C NMR (75 MHz, MeOD): δ = 71.76, 60.05, 58.76, 56.54, 56.29, 56.10, 54.85, 48.76, 47.34, 42.70, 42.62, 40.58, 39.98, 39.49, 38.92, 36.15, 35.76, 35.63, 35.28, 31.03, 30.40, 30.21, 28.19, 27.87, 25.93, 24.34, 23.79, 22.78, 22.52, 21.05, 18.64, 12.12. $C_{32}H_{60}N_2O$; MS (ESI) m/z = 489.5 $[M+H]^+$

(3β,6β)-6-[(3-aminopropyl)amino)propyl)amino]cholestan-3-ol 4e: Purification by column chromatography (silica gel; CH_2Cl_2/ MeOH/ NH_4OH(32%), 7:3:1) afforded a pale yellow solid in 43% yield; – ^{1}H NMR (300 MHz, MeOD): δ 3.57 (d, *J* = 13.30 Hz, 1H), 2.85 (m, 5H), 2.63 (m, 3H), 2.05 (m, 2H), 1.71 (m, 14H), 1.38 (m, 9H), 1.14 (dq, *J* = 5.40, 20.60 Hz, 8H), 0.92 (m, 13H), 0.74 (h, *J* = 4.73, 5.20 Hz, 4H)..– ^{13}C NMR (75 MHz, MeOD): δ = 72.30, 60.64, 57.71, 57.44, 56.04, 48.03, 47.45, 44.17, 43.89, 42.06, 41.31, 40.88, 40.70, 40.13, 39.41, 37.74, 37.36, 37.14, 36.69, 36.53, 32.20, 31.74, 31.52, 31.35, 29.32, 29.15, 25.39, 24.95, 23.20, 22.95, 22.18, 19.23, 16.64, 12.66. $C_{33}H_{63}N_3O$; MS (ESI) m/z = 518.4 $[M+H]^+$
4-aminobutyl)amino)propyl)amino
(3β,6β)-6-[(4-aminobutyl)amino)propyl)amino]cholestan-3-ol 4g (mixture of isomers): Purification by column chromatography (silica gel; CH_2Cl_2/ MeOH/ NH_4OH(32%), 7:3:1) afforded a pale yellow solid in 28% yield; – ^{1}H NMR (300 MHz, MeOD): δ = 3.58 (m, 1H), 2.82 (m, 2H), 2.73 (s, 6H), 2.64 (dd, *J* = 6.51, 12.97 Hz, 1H), 2.07 (td, *J* = 9.83, 15.41, 18.28 Hz, 2H), 1.87 (m, 1H), 1.78 (m, 1H), 1.66 (tdd, *J* = 4.89, 9.61, 14.66 Hz, 4H), 1.55 (h, *J* = 6.48 Hz, 2H), 1.41 (dd, *J* = 10.47, 14.51 Hz, 4H), 1.39 (s, 10H), 1.32 (d, *J* = 13.56 Hz, 1H), 1.15 (ddp, *J* = 5.44, 6.70, 10.65, 16.20 Hz, 9H), 1.01 (m, 4H), 0.93 (m, 7H), 0.89 (m, 5H), 0.74 (t, *J* = 7.02 Hz, 6H). – ^{13}C NMR (75 MHz, MeOD): δ = 72.39, 71.45, 71.11, 67.02, 60.58, 60.36, 57.87, 57.70, 57.52, 56.16, 56.05, 54.93, 53.63, 45.30, 44.17, 43.95, 43.89, 42.05, 41.35, 40.88, 40.70, 40.17, 39.40, 37.74, 37.37, 37.31, 37.15, 37.05, 36.71, 36.16, 32.23, 31.73, 31.52, 30.69, 30.58, 29.34, 29.18, 29.15, 28.08, 25.38, 25.01, 24.95, 24.19, 23.24, 23.21, 23.00, 22.96, 22.61, 22.21, 21.89, 19.26, 19.19, 16.68, 13.44, 12.68, 12.43. $C_{34}H_{65}N_3O$; MS (ESI) m/z = 532.6 $[M+H]^+$

(3β,6β)-6-[(2-((2-((2-aminoethyl)amino)ethyl)amino)ethyl)amino]cholestan-3-ol 4h: Purification by column chromatography (silica gel; CH_2Cl_2/ MeOH/ NH_4OH(32%), 7:3:1) afforded a pale yellow solid in 38% yield; – ^{1}H NMR (300 MHz, MeOD): δ = 4.88 (s, 9H), 4.03 (s, 1H), 3.45 (m, 5H), 3.15 (m, 2H), 2.92 (m, 3H), 2.76 (m, 2H), 2.56 (m, 4H), 2.02 (m,

1H), 1.86 (m, 1H), 1.65 (m, 2H), 1.53 (dq, *J* = 6.60, 13.18 Hz, 2H), 1.40 (t, *J* = 7.88 Hz, 3H), 1.37 (d, *J* = 4.23 Hz, 1H), 1.34 (s, 2H), 1.14 (tdd, *J* = 6.21, 9.76, 15.32 Hz, 6H), 1.00 (q, *J* = 5.70 Hz, 2H), 0.93 (d, *J* = 6.30 Hz, 3H), 0.88 (dd, *J* = 1.57, 6.59 Hz, 7H), 0.72 (qd, *J* = 2.62, 6.99, 7.68 Hz, 4H). ^{13}C NMR (75 MHz, MeOD): δ = 72.36, 57.91, 57.68, 57.60, 56.13, 55.30, 53.87, 53.78, 50.21, 50.12, 50.08, 46.98, 43.87, 41.79, 41.34, 41.14, 40.68, 40.23, 39.35, 37.95, 37.36, 37.12, 36.71, 36.20, 35.99, 32.21, 31.72, 29.33, 29.13, 28.08, 25.36, 24.95, 23.24, 22.99, 22.21, 19.29, 16.70, 12.69. $C_{33}H_{64}N_4O$; MS (ESI) m/z = 533.6 $[M+H]^+$

(3β,6β)-6-[(2-((2-((2-aminoethyl)amino)ethyl)amino)ethyl)amino)ethyl)amino]cholestan-3-ol 4i: Purification by column chromatography (silica gel; CH_2Cl_2/ MeOH/ NH_4OH(32%), 7:3:1) afforded a pale yellow solid in 51% yield; – ^{1}H NMR (300 MHz, MeOD): δ = 2.72 (dt, *J* = 6.15, 23.21 Hz, 6H), 2.52 (tdd, *J* = 6.15, 13.18, 24.10 Hz, 4H), 1.89 (m, 2H), 1.69 (m, 7H), 1.54 (ddd, *J* = 3.23, 6.49, 11.31 Hz, 4H), 1.40 (m, 5H), 1.33 (ddd, *J* = 5.08, 8.85, 21.44 Hz, 4H), 1.15 (m, 4H), 1.14 (s, 8H), 0.94 (m, 16H), 0.89 (m, 4H), 0.75 (d, *J* = 2.69 Hz, 4H). ^{13}C NMR (75 MHz, MeOD): δ = 72.48, 61.25, 60.52, 60.40, 57.78, 57.55, 56.27, 54.22, 54.14, 43.93, 43.90, 41.41, 40.75, 40.72, 40.30, 38.96, 37.40, 37.22, 37.17, 36.85, 36.74, 32.24, 31.75, 30.30, 29.35, 29.19, 29.17, 25.41, 25.06, 24.99, 23.27, 23.22, 23.03, 22.98, 22.27, 19.35, 19.28, 17.15, 16.90, 12.80. $C_{35}H_{69}N_5O$; MS (ESI) m/z = 576.6 $[M+H]^+$

14-amino-3,6,9,12-tetraazatetradecyl)

(3β,6β)-6-[(14-amino-3,6,9,12-tetraazatetradecyl)amino]cholestan-3-ol 4j: Purification by column chromatography (silica gel; CH_2Cl_2/ MeOH/ NH_4OH(32%), 7:3:1) afforded a pale yellow solid in 34% yield; – ^{1}H NMR (300 MHz, MeOD): δ δ 3.58 (dq, *J* = 4.47, 5.07, 11.64 Hz, 1H), 3.47 (s, 2H), 2.88 (dd, *J* = 2.10, 5.04 Hz, 2H), 2.82 (m, 3H), 2.79 (s, 5H), 2.77 (m, 5H), 2.60 (dd, *J* = 3.67, 16.05 Hz, 4H), 2.55 (s, 2H), 2.51 (m, 6H), 2.06 (d, *J* = 12.13 Hz, 2H), 1.90 (m, 2H), 1.75 (dd, *J* = 13.09, 25.12 Hz, 4H), 1.56 (m, 3H), 1.44 (s, 2H), 1.40 (s, 5H), 1.34 (m, 1H), 1.08 (m, 20H), 0.92 (dt, *J* = 1.26, 6.62 Hz, 9H), 0.85 (d, *J* = 7.24 Hz, 2H), 0.76 (m, 5H). ^{13}C NMR (75 MHz, MeOD): δ = 70.64, 59.15, 58.14, 56.11, 51.11, 51.71, 48.89, 48.15, 47.04, 46.81, 46.33, 45.67, 45.57, 45.47, 45.36, 42.74, 41.96, 39.90, 39.40, 36.89, 36.22, 35.78, 34.39, 33.32, 32.98, 32.87, 31.15, 28.88, 28.21, 24.30, 24.00, 23.66, 20.29, 18.25, 16.94, 12.96. $C_{37}H_{74}N_6O$; MS (ESI) m/z = 619.5 $[M+H]^+$

(3β,6β)-6-[((3-((3-aminopropyl)(methyl)amino)propyl)amino]cholestan-3-ol 4k: Purification by column chromatography (silica gel; CH_2Cl_2/ MeOH/ NH_4OH(32%), 7:3:1)

afforded a pale yellow solid in 48% yield; – ^{1}H NMR (300 MHz, MeOD): δ = 3.57 (tt, *J* = 4.67, 10.38 Hz, 2H), 2.66 (m, 6H), 2.45 (m, 6H), 2.26 (s, 4H), 2.05 (m, 2H), 1.89 (m, 2H), 1.72 (m, 4H), 1.67 (m, 6H), 1.50 (m, 2H), 1.38 (m, 6H), 1.18 (m, 4H), 1.14 (d, *J* = 4.60 Hz, 2H), 0.93 (m, 22H), 0.75 (m, 4H). ^{13}C NMR (75 MHz, MeOD): δ = 72.44, 60.42, 57.81, 57.58, 57.24, 56.53, 56.30, 43.92, 42.50, 41.45, 41.00, 40.75, 40.30, 37.46, 37.35, 37.20, 36.79, 36.70, 32.23, 31.74, 30.73, 29.40, 29.17, 28.12, 25.44, 25.06, 23.30, 23.06, 22.28, 19.39, 16.73, 12.73. $C_{34}H_{65}N_3O$; MS (ESI) m/z = 532.4 $[M+H]^+$

(3β,6β)-6-[(2-(bis(2-aminoethyl)amino)ethyl)amino]cholestan-3-ol 4l: Purification by column chromatography (silica gel; CH_2Cl_2/ MeOH/ NH_4OH(32%), 7:3:1) afforded a pale yellow solid in 49% yield; – ^{1}H NMR (300 MHz, MeOD): δ 3.38 (m, 5H), 3.14 (s, 2H), 3.05 (m, 2H), 2.71 (m, 10H), 1.91 (m, 10H), 1.61 (m, 10H), 1.37 (m, 10H), 1.15 (qd, *J* = 4.88, 10.44, 13.33 Hz, 6H), 0.92 (m, 8H), 0.73 (m, 4H). – ^{13}C NMR (75 MHz, MeOD): δ = 72.35, 67.09, 60.63, 57.72, 57.49, 57.25, 56.13, 55.11, 54.99, 43.94, 43.89, 41.89, 41.35, 40.69, 40.14, 39.58, 37.36, 37.14, 36.71, 32.23, 31.75, 31.33, 29.33, 29.15, 25.40, 24.95, 24.25, 23.20, 22.99, 22.95, 22.20, 19.24, 16.70, 12.64, 12.58. $C_{33}H_{64}N_4O$; MS (ESI) m/z = 533.4 $[M+H]^+$

(3β,6β)-6-[(2-(4-(2-aminoethyl)piperazin-1-yl)ethyl)amino]cholestan-3-ol 4m: Purification by column chromatography (silica gel; CH_2Cl_2/ MeOH/ NH_4OH(32%), 7:3:1) afforded a pale yellow solid in 53% yield; – ^{1}H NMR (300 MHz, MeOD): δ 3.47 (s, 2H), 2.94 (qd, *J* = 4.64, 10.43, 11.09 Hz, 2H), 2.53 (s, 9H), 2.51 (m, 5H), 2.05 (m, 2H), 1.80 (m, 6H), 1.53 (m, 2H), 1.40 (m, 5H), 1.11 (m, 14H), 0.94 (dd, *J* = 2.35, 6.85 Hz, 4H), 0.88 (m, 8H), 0.73 (dd, *J* = 3.49, 10.60 Hz, 4H).– ^{13}C NMR (75 MHz, MeOD): δ = 72.22, 66.73, 61.91, 61.07, 58.47, 57.69, 57.59, 57.46, 56.98, 55.93, 54.16, 54.10, 53.85, 53.69, 43.97, 43.90, 41.29, 40.69, 40.35, 39.98, 37.37, 37.14, 36.62, 36.48, 36.05, 35.72, 32.78, 32.13, 31.74, 31.24, 29.77, 28.73, 27.95, 26.24, 25.34, 24.96, 23.22, 23.03, 19.27, 16.74, 12.76. $C_{35}H_{66}N_4O$; MS (ESI) m/z = 559.3 $[M+H]^+$

(3β,6β)-6-[(2-(piperazin-1-yl)ethyl)amino]cholestan-3-ol 4n: Purification by column chromatography (silica gel; CH_2Cl_2/ MeOH/ NH_4OH(32%), 7:3:1) afforded a pale yellow solid in 76% yield; – ^{1}H NMR (300 MHz, MeOD): δ = δ 3.51 (m, 1H), 2.96 (m, 4H), 2.83 (m, 2H), 2.58 (m, 6H), 1.38 (m, 30H), 0.90 (dd, *J* = 1.54, 6.68 Hz, 4H), 0.81 (d, *J* = 6.70 Hz, 4H), 0.65 (m, 7H).– ^{13}C NMR (75 MHz, MeOD): δ = 71.68, 59.08, 58.12, 56.27, 56.06, 54.81, 54.07, 53.91, 53.80, 47.32, 45.91, 45.42, 42.62, 39.94, 39.46, 38.99, 36.12, 35.74, 35.61, 35.16, 31.57,

30.42, 28.17, 27.96, 25.95, 24.33, 23.77, 22.76, 22.52, 21.03, 18.64, 16.25, 12.17. $C_{33}H_{61}N_3O$; MS (ESI) m/z = 530.2 M+H]$^+$

(3β,6β)-6-[(3-(1H-imidazol-1-yl)propyl)amino]cholestan-3-ol 4o: Purification by column chromatography (silica gel; CH_2Cl_2/ MeOH/ NH_4OH(32%), 7:3:1) afforded a pale yellow solid in 64% yield; – ^{1}H NMR (300 MHz, MeOD): δ = 3.60 (m, 2H), 3.56-3.63 (m, 3H), 0.56-2.70 (m, 50H) – ^{13}C NMR (75 MHz, MeOD): δ = 137.14, 128.95, 118.92, 71.35, 58.93, 56.21, 55.90, 54.67, 47.14, 44.86, 44.50, 42.56, 39.84, 39.41, 38.90, 38.52, 36.07, 35.82, 35.68, 35.61, 31.67, 31.44, 30.40, 28.10, 27.91, 24.27, 23.72, 22.72, 22.47, 20.97, 18.59, 16.27, 12.06. $C_{33}H_{57}N_3O$; MS (ESI) m/z = 512.7 [M+H]$^+$

(3β,6β)-6-[(3-(4-(3-aminopropyl)piperazin-1-yl)propyl)amino]cholestan-3-ol 4p: Purification by column chromatography (silica gel; CH_2Cl_2/ MeOH/ NH_4OH(32%), 7:3:1) afforded a pale yellow solid in 53% yield; – ^{1}H NMR (300 MHz, MeOD): δ = 3.56 (m, 1H), 2.84 (m, 3H), 2.72 (s, 1H), 2.63 (d, *J* = 3.08 Hz, 1H), 2.57 (s, 1H), 2.53 (s, 11H), 2.47 (dd, *J* = 2.46, 6.75 Hz, 2H), 2.44 (s, 6H), 2.01 (m, 2H), 1.87 (m, 1H), 1.74 (m, 4H), 1.69 (s, 9H), 1.53 (m, 2H), 1.37 (m, 5H), 1.16 (td, *J* = 4.30, 8.19 Hz, 5H), 1.02 (d, *J* = 5.21 Hz, 1H), 0.91 (m, 10H), 0.73 (s, 3H), 0.69 (s, 1H). – ^{13}C NMR (75 MHz, MeOD): δ = 72.36, 60.74, 58.44, 57.74, 57.52, 57.11, 56.15, 54.13, 53.79, 48.51, 43.90, 41.38, 40.71, 40.57, 40.15, 37.40, 37.16, 36.73, 32.21, 31.76, 29.36, 29.15, 27.50, 27.15, 25.42, 25.00, 23.26, 23.02, 22.24, 19.32, 16.83, 12.76. $C_{37}H_{70}N_4O$; MS (ESI) m/z = 587.5 [M+H]$^+$

6(3β,6β)-6-[(3-(bis(3-aminopropyl)amino)propyl)amino]cholestan-3-ol 4q: Purification by column chromatography (silica gel; CH_2Cl_2/ MeOH/ NH_4OH(32%), 7:3:1) afforded a pale yellow solid in 47% yield; – ^{1}H NMR (300 MHz, MeOD): δ = 4.87 (s, 7H), 3.55 (ddt, *J* = 5.30, 9.97, 13.15 Hz, 1H), 3.26 (m, 1H), 3.09 (q, *J* = 6.89 Hz, 1H), 2.85 (q, *J* = 6.70 Hz, 1H), 2.76 (t, *J* = 7.02 Hz, 2H), 2.63 (qd, *J* = 5.45, 9.38, 11.89 Hz, 1H), 2.52 (dt, *J* = 7.20, 13.59 Hz, 5H), 2.01 (m, 1H), 1.85 (m, 1H), 1.70 (dd, *J* = 6.59, 14.65 Hz, 6H), 1.64 (s, 5H), 1.51 (h, *J* = 6.80, 7.22 Hz, 2H), 1.41 (tt, *J* = 4.59, 9.27 Hz, 5H), 1.36 (m, 1H), 1.28 (s, 1H), 1.12 (m, 7H), 0.99 (s, 3H), 0.93 (d, *J* = 6.25 Hz, 4H), 0.87 (dd, *J* = 1.56, 6.67 Hz, 6H), 0.72 (s, 3H), 0.70 (m, 1H). – ^{13}C NMR (75 MHz, MeOD): δ = 72.37, 67.04, 61.42, 60.40, 57.76, 57.70, 57.54, 56.21, 53.29, 53.25, 52.73, 52.66, 43.95, 43.90, 41.41, 40.72, 40.59, 40.23, 37.42, 37.17, 36.74, 36.20, 32.23,

31.72, 29.38, 29.15, 28.73, 25.42, 25.02, 24.98, 23.30, 23.05, 22.26, 21.95, 19.38, 16.69, 12.74. $C_{36}H_{70}N_4O$; MS (ESI) m/z = 575.4 $[M+H]^+$

(3β,6β)-6-[(3-((4-((3-aminopropyl)amino)butyl)amino)propyl)amino]cholestan-3-methoxy 4r: by column chromatography (silica gel; CH_2Cl_2/ MeOH/ NH_4OH(32%), 7:3:1) afforded a pale yellow solid in 49% yield; – ^{1}H NMR (300 MHz, MeOD): δ = 3.68 (m, 1H), 3.28 (d, *J* = 1.65 Hz, 2H), 2.97 (m, 4H), 2.63 (m, 4H), 2.58 (tt, *J* = 5.04, 6.22 Hz, 2H), 2.41 (m, 4H), 1.40 (m, 40H), 0.952 (dd, *J* = 1.54, 6.68 Hz, 4H), 0.84 (d, *J* = 6.70 Hz, 4H), 0.68 (m, 6H). – ^{13}C NMR (75 MHz, MeOD): δ = 70.74, 60.75, 57.15, 56.92, 54.68, 53.79, 51.86, 48.65, 46.46, 45.87, 42.94, 40.56, 39.41, 36.89, 36.42, 35.77, 34.38, 33.98, 32.87, 32.54, 30.95, 29.28, 28.12, 27.24, 24.98, 24.05, 22.46, 22.29, 20.02, 15.96, 11.47. $C_{38}H_{74}N_4O$; MS (ESI) m/z = 603.4 $[M+H]^+$

^{13}C and ^{1}H NMR spectra of compounds 4a-4s

^{13}C and ^{1}H NMR spectra of 4a

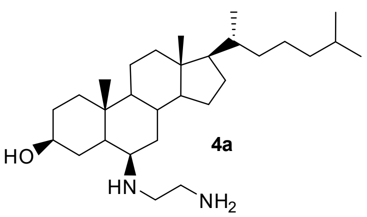

4a

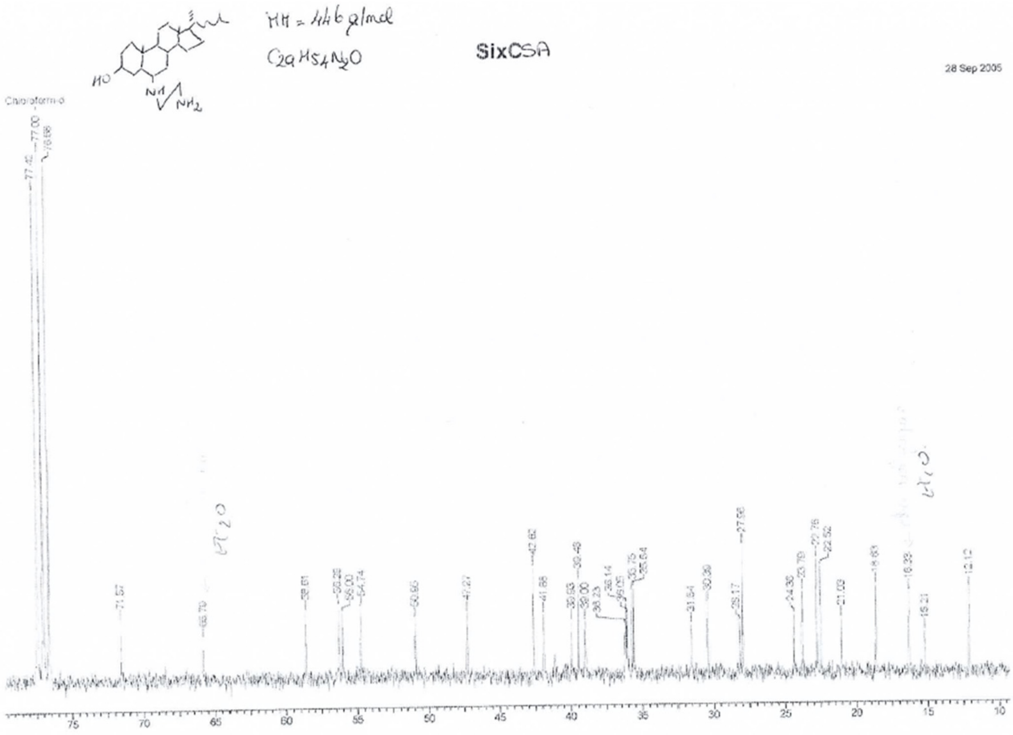

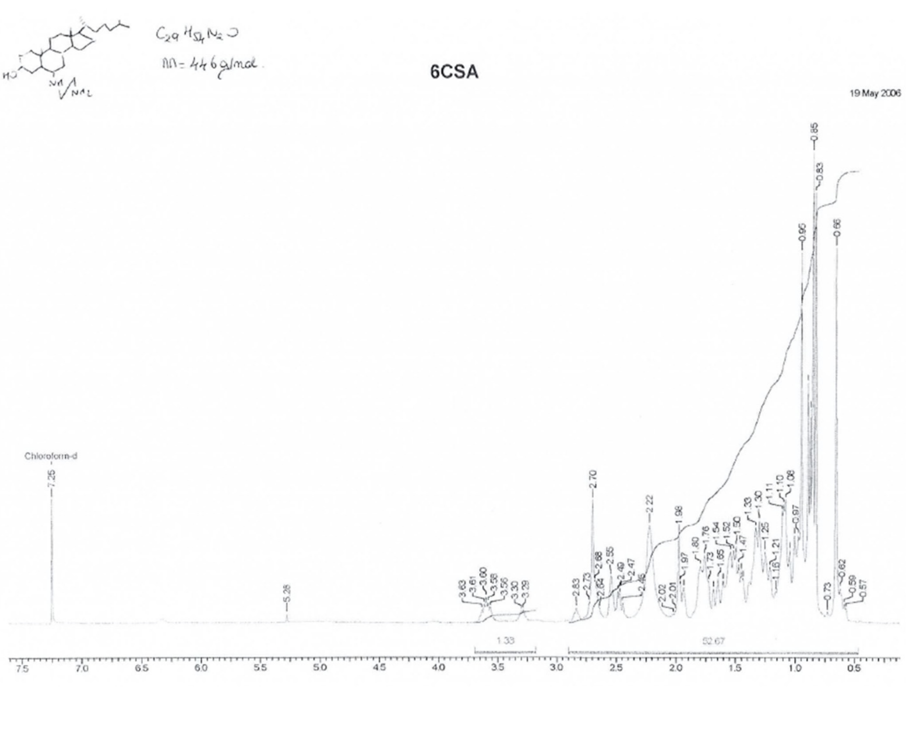

^{13}C and ^{1}H NMR spectra of 4b

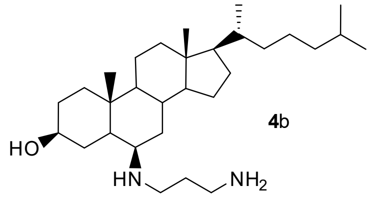

4b

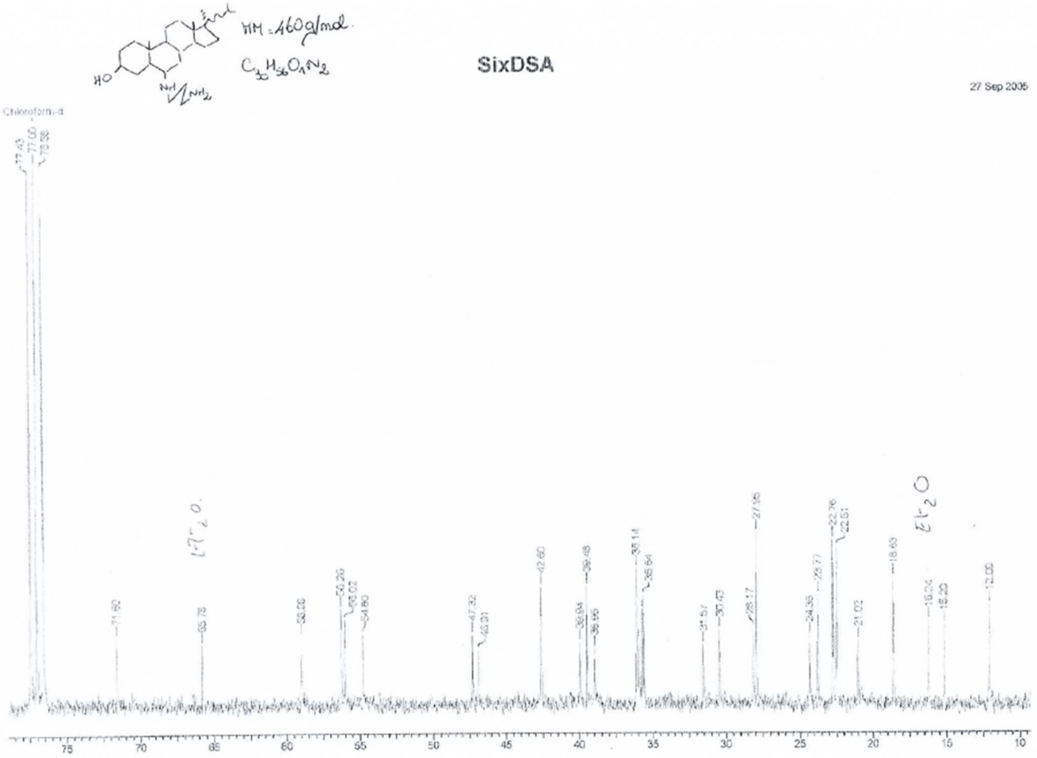

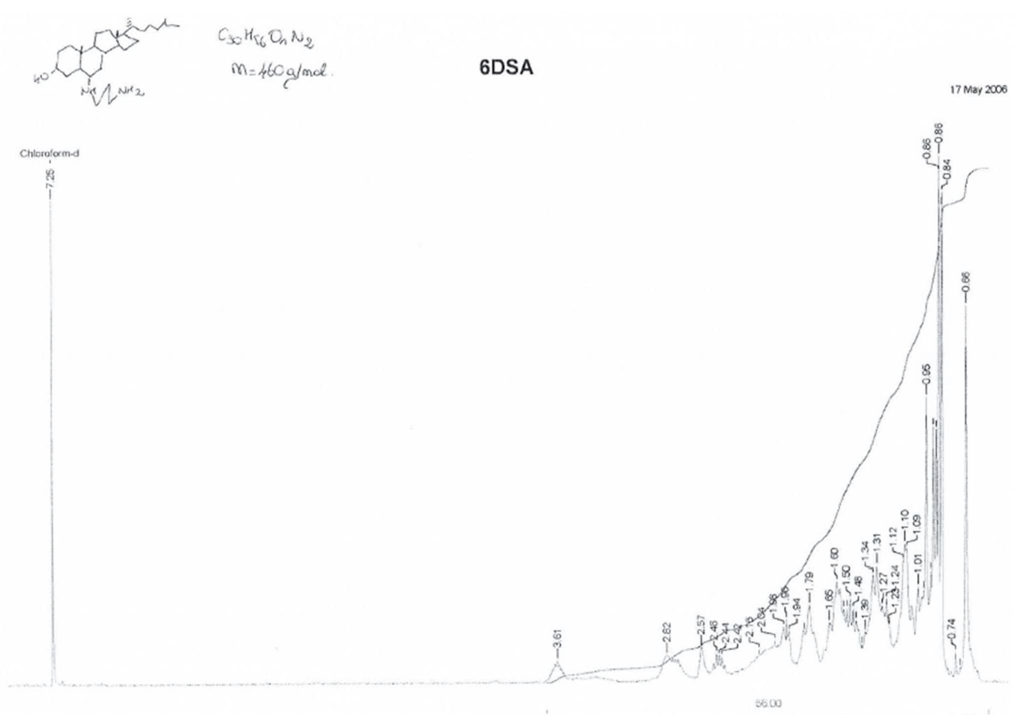

^{13}C and ^{1}H NMR spectra of 4c

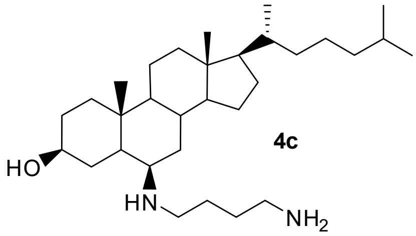

4c

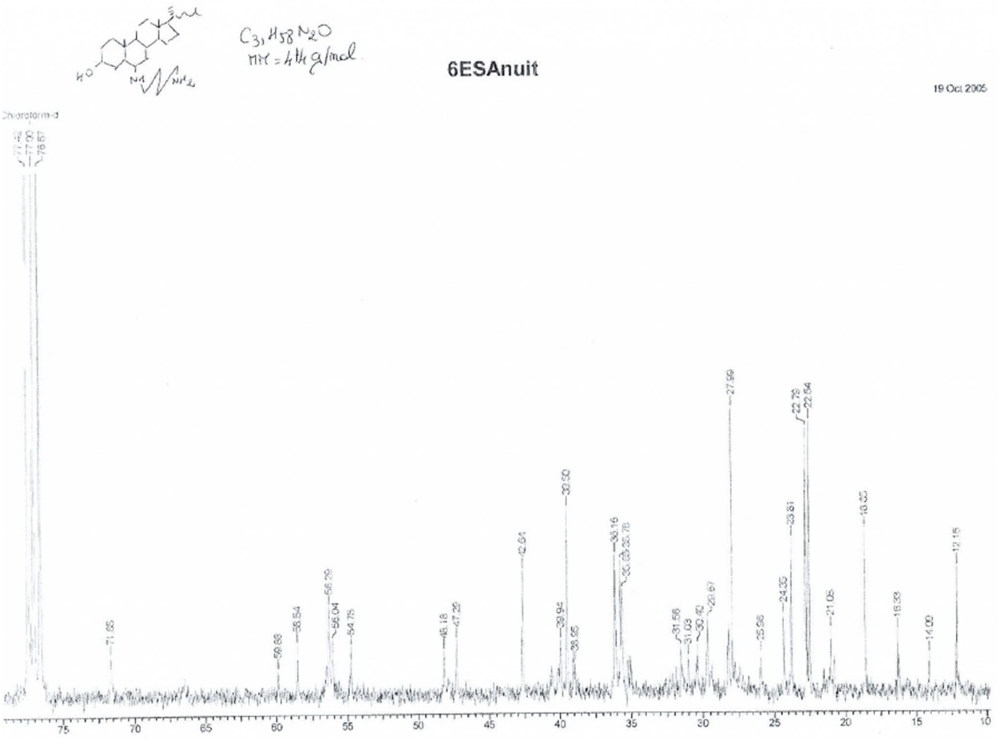

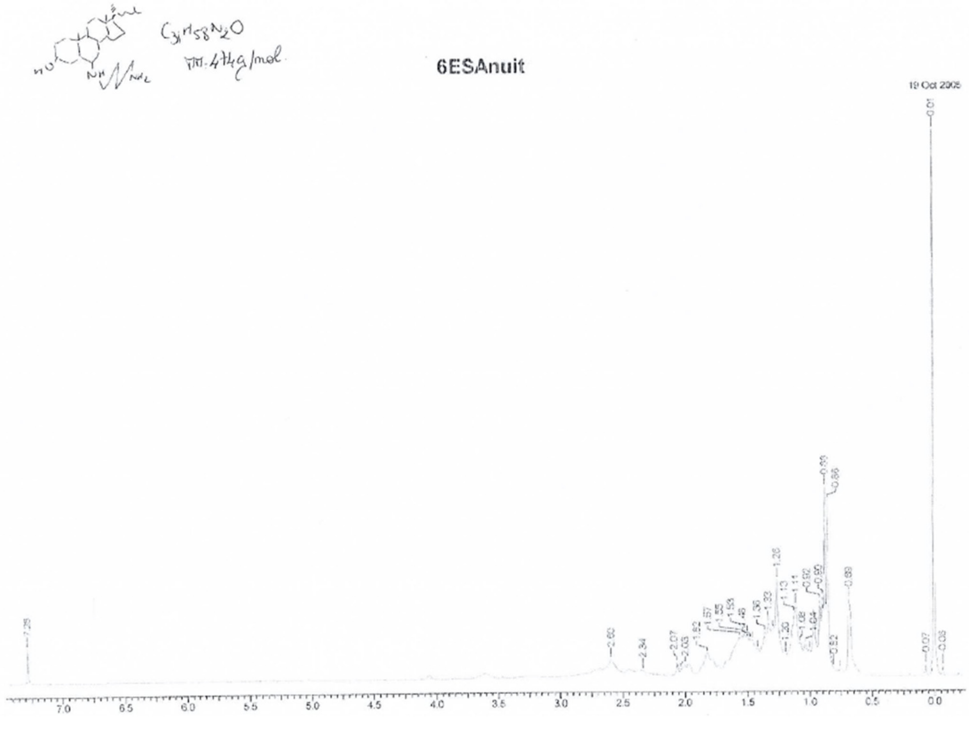

^{13}C and ^{1}H NMR spectra of 4d

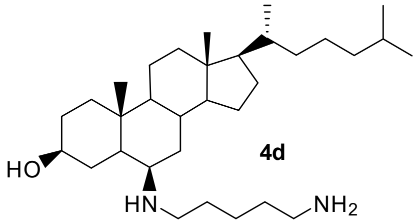

4d

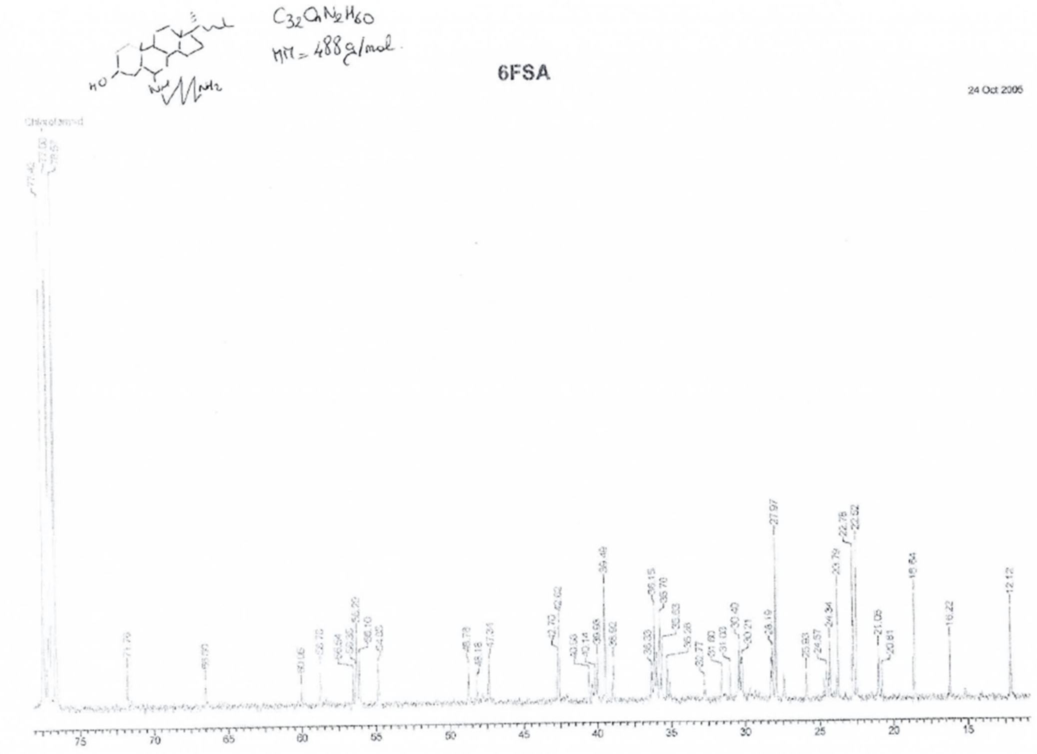

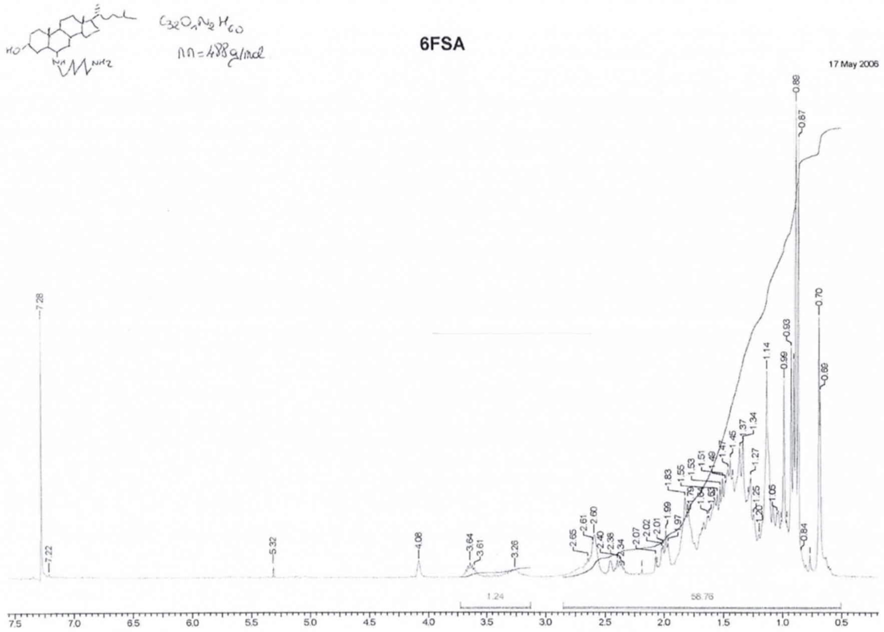

^{13}C and ^{1}H NMR spectra of 4e

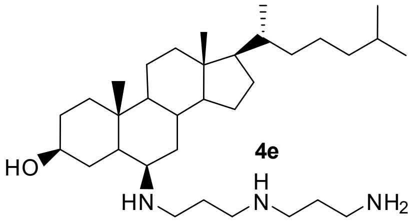

4e

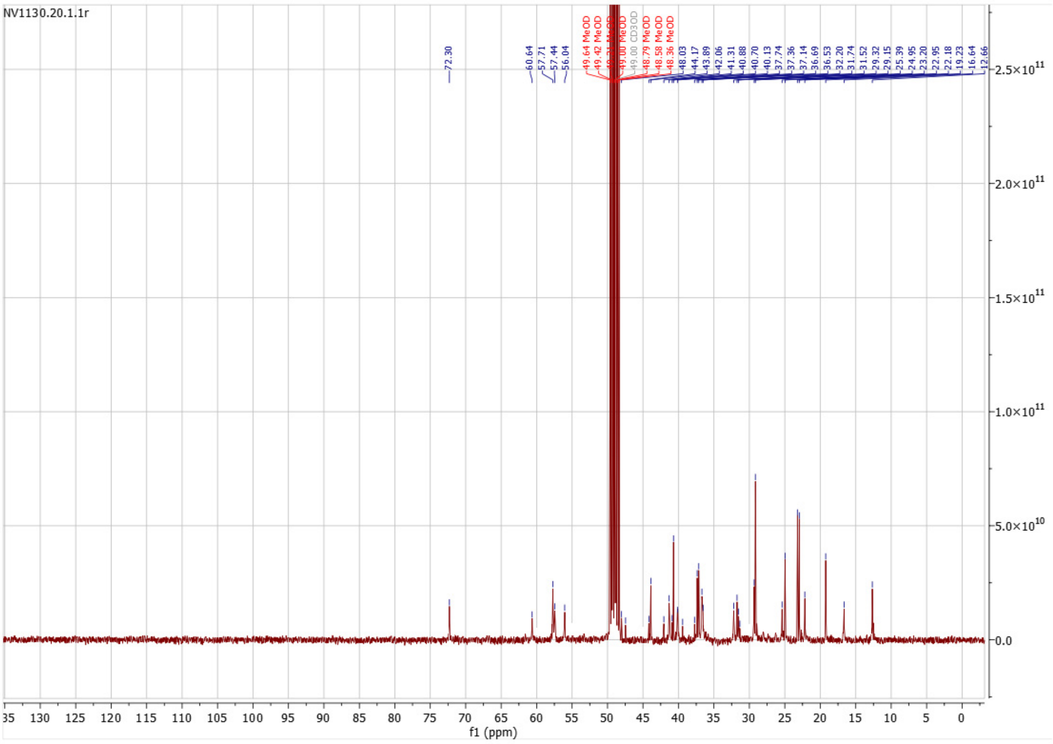

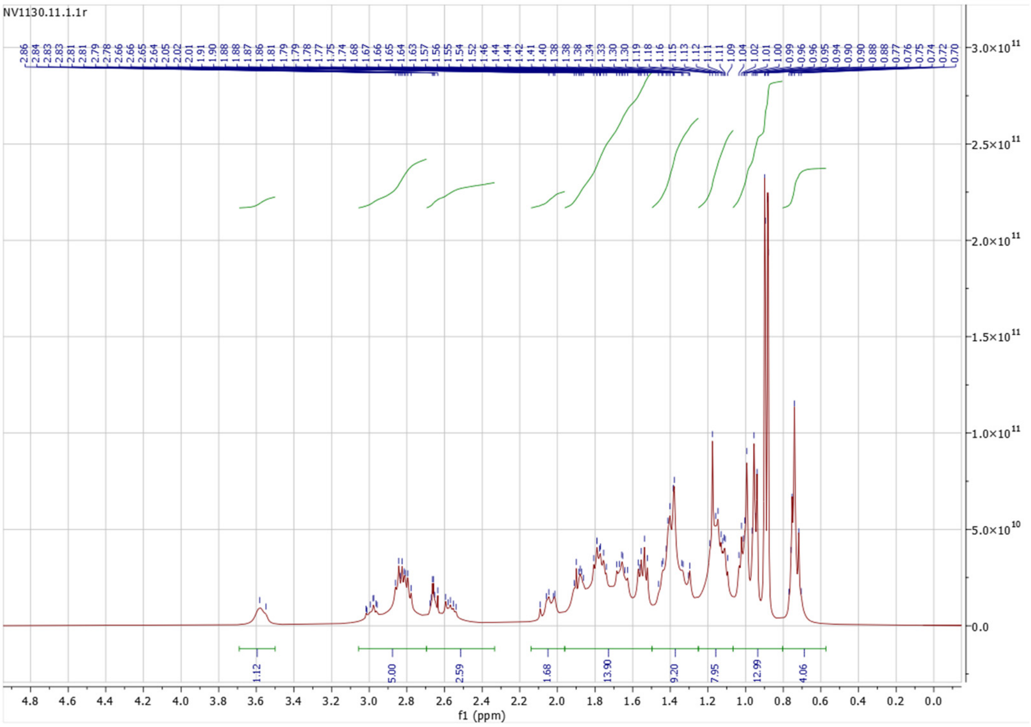

^{13}C and ^{1}H NMR spectra of 4f

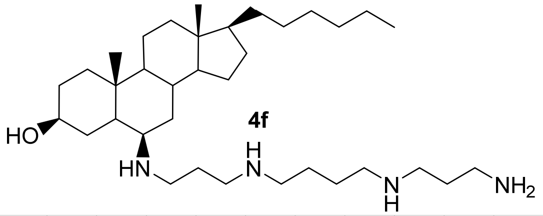

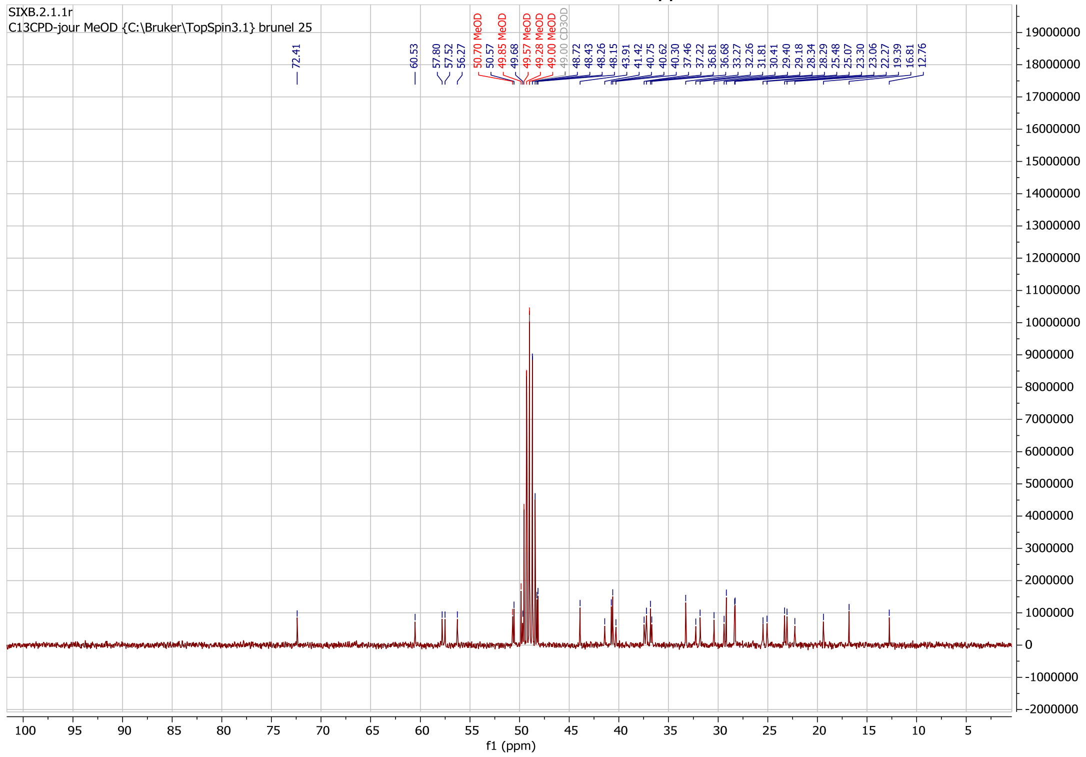

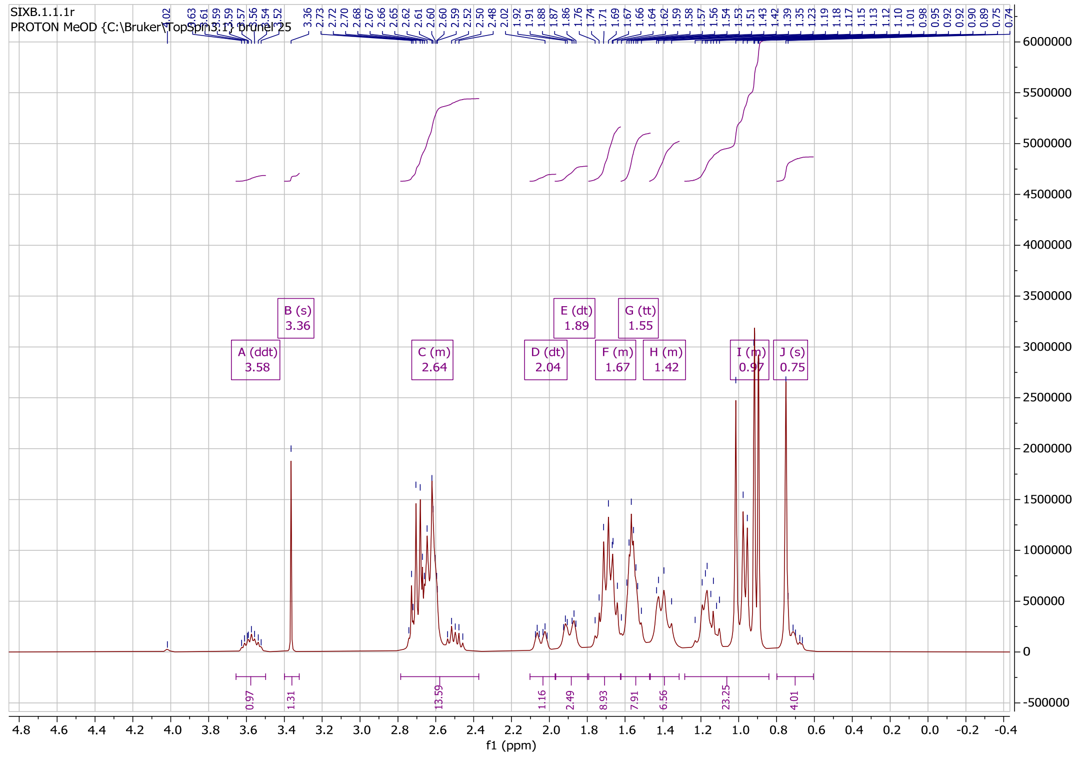

^{13}C and ^{1}H NMR spectra of 4g

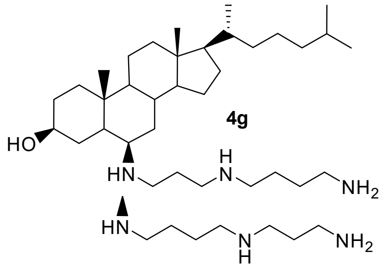

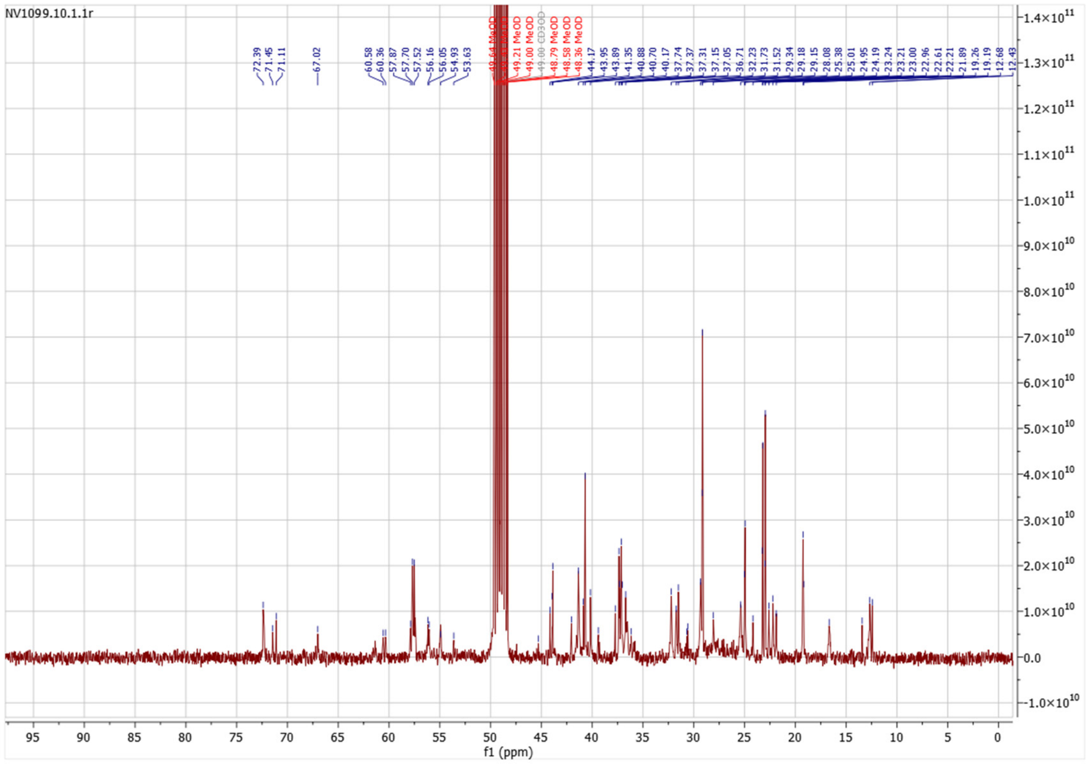

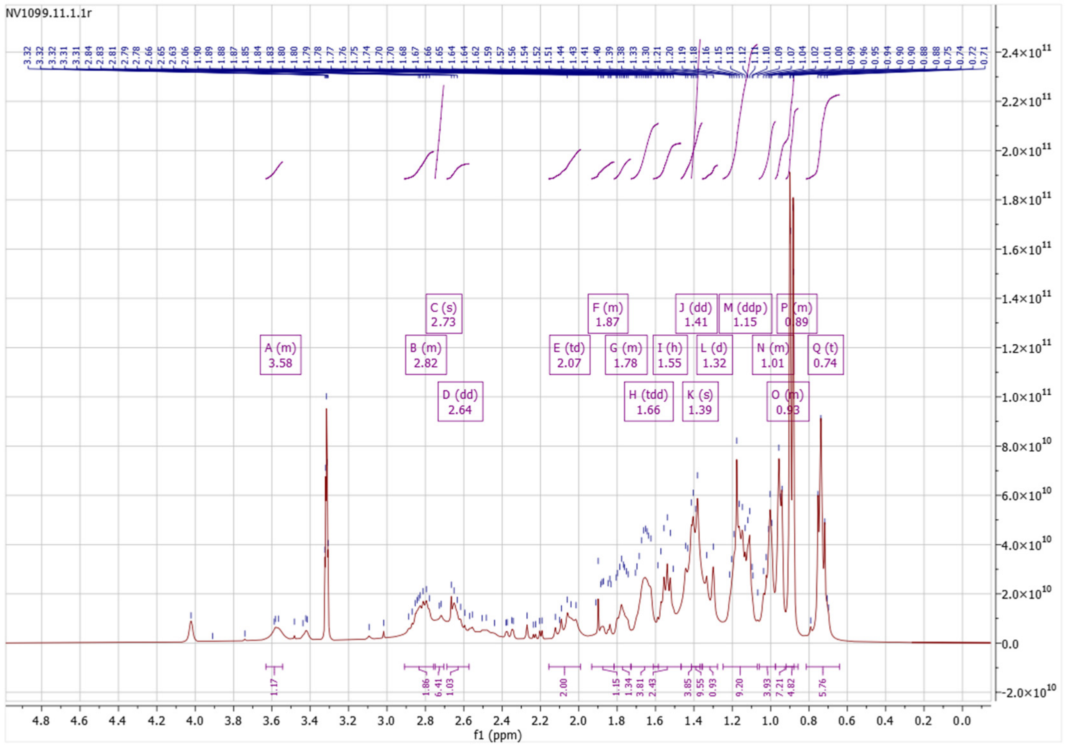

^{13}C and ^{1}H NMR spectra of 4h

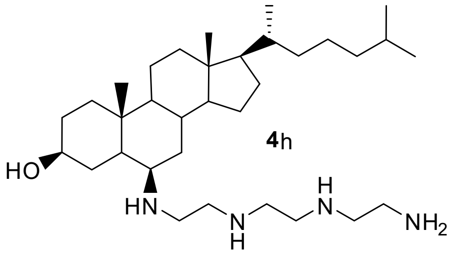

4h

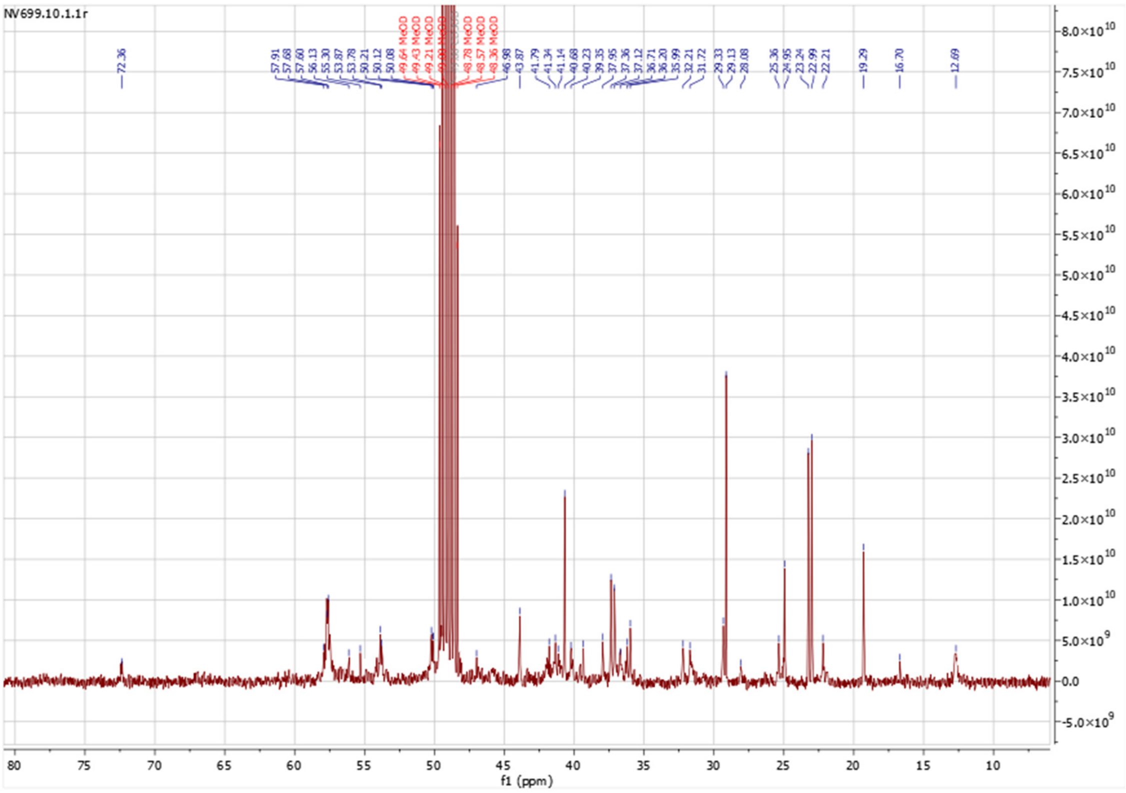

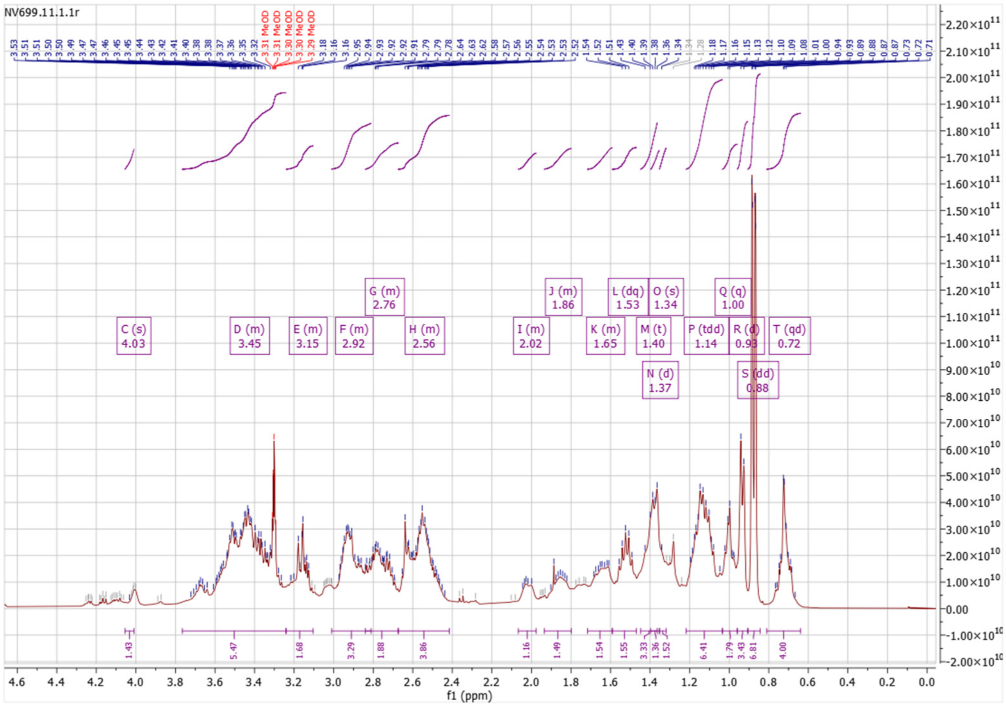

^{13}C and ^{1}H NMR spectra of 4i

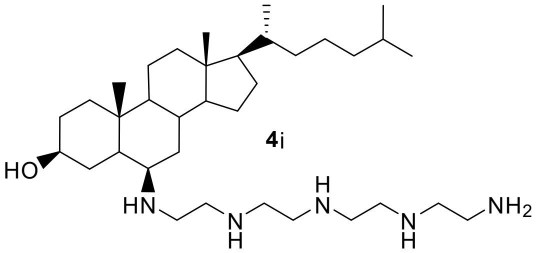

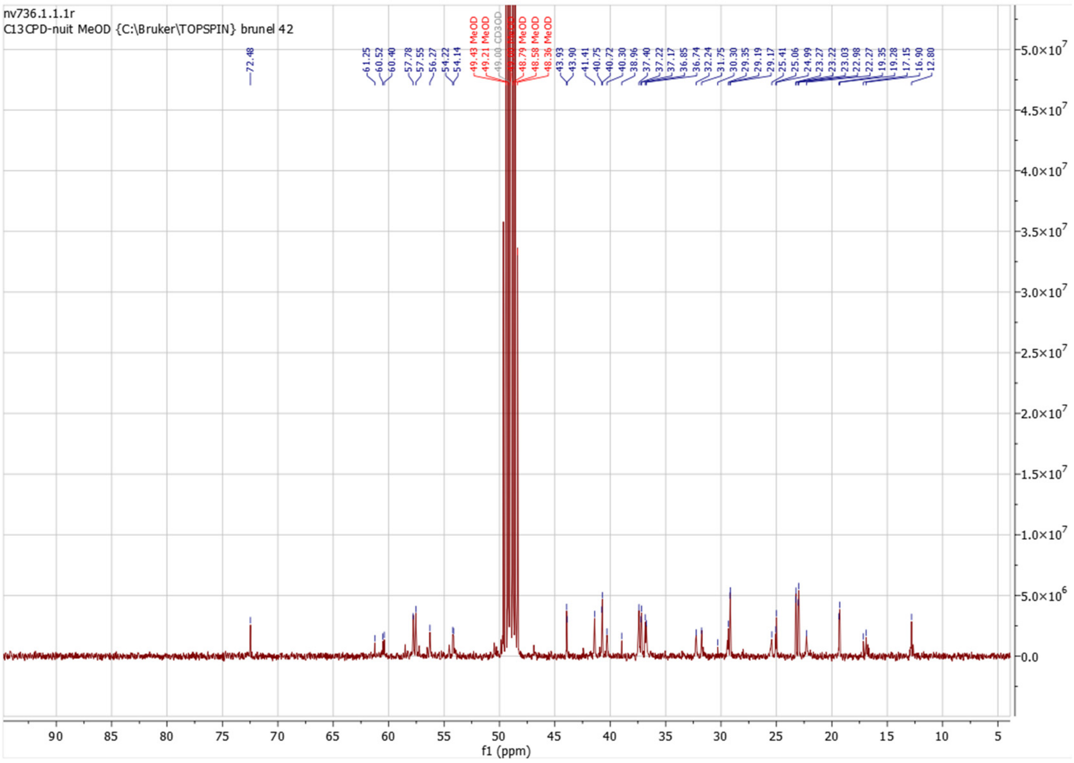

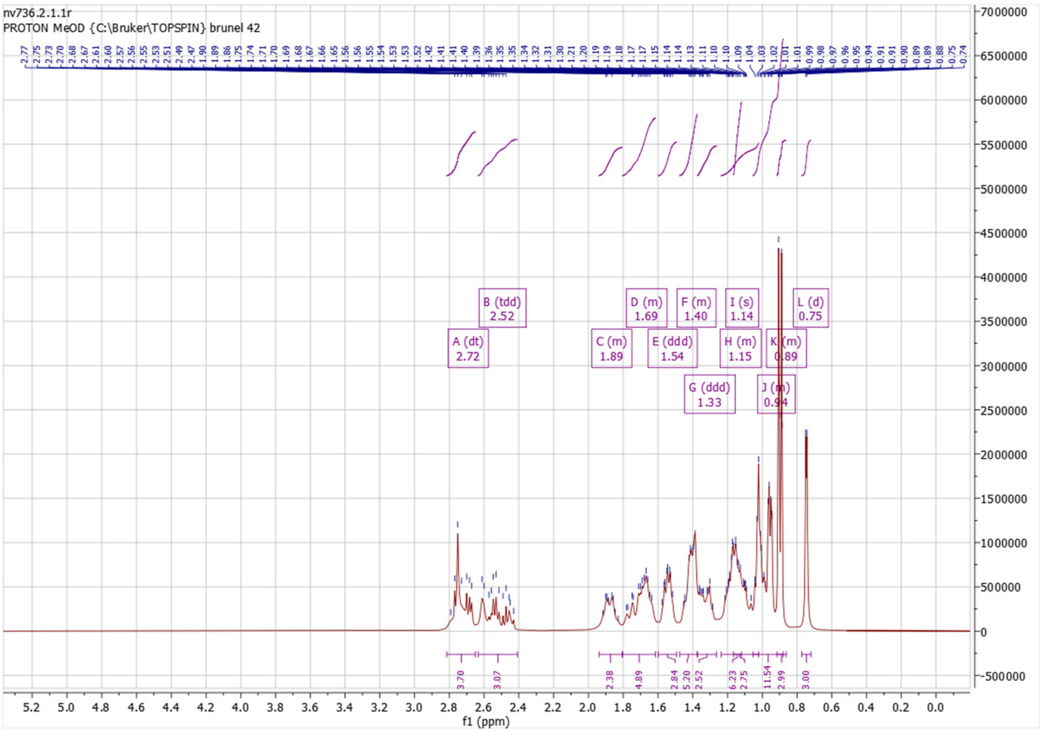

^{13}C and ^{1}H NMR spectra of 4j

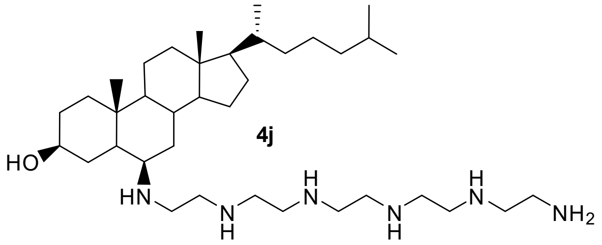

4j

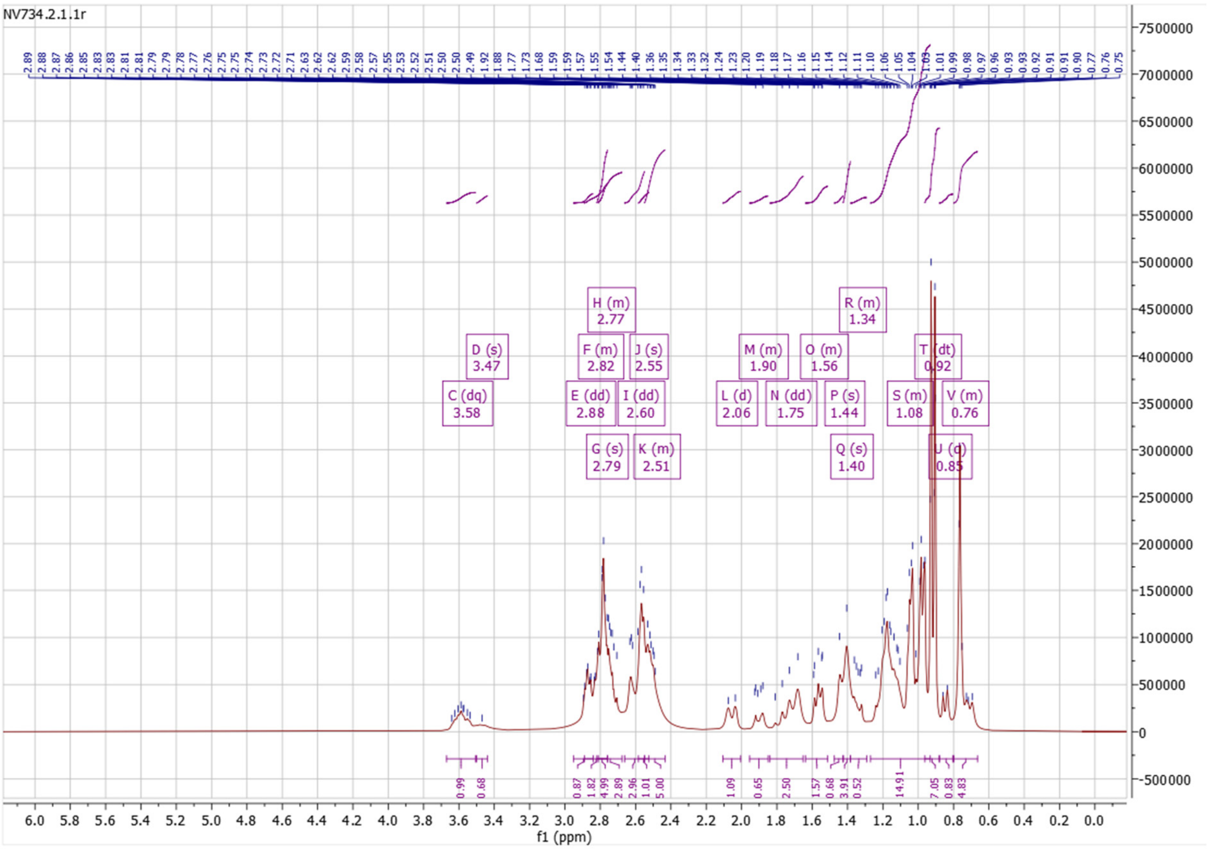

^{13}C and ^{1}H NMR spectra of 4k

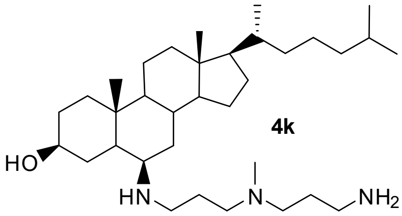

4k

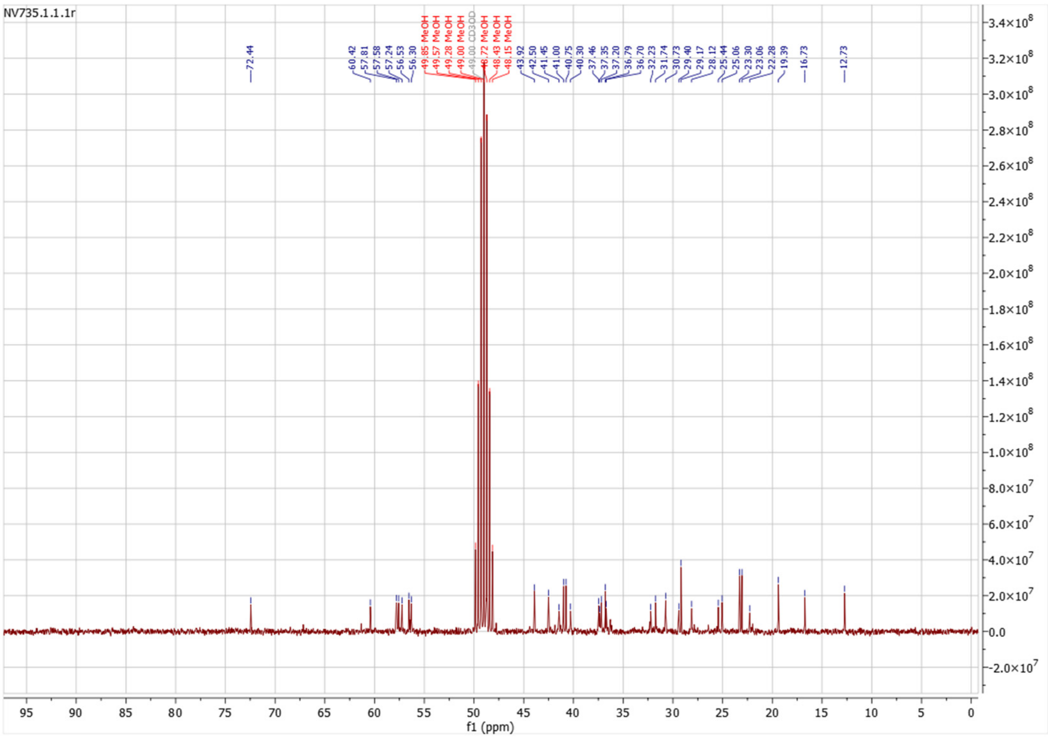

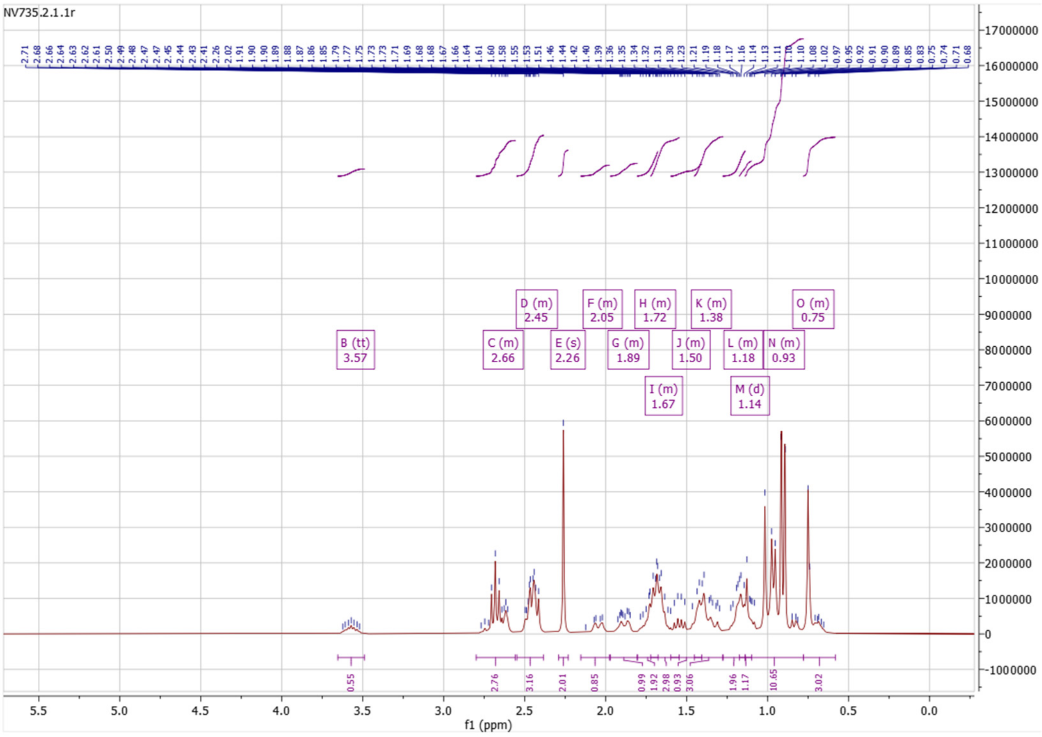

^{13}C and ^{1}H NMR spectra of 4l

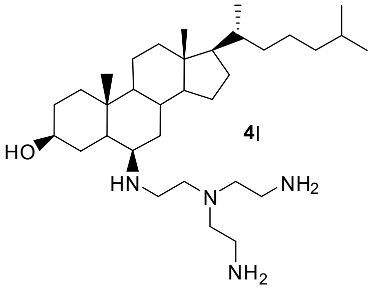

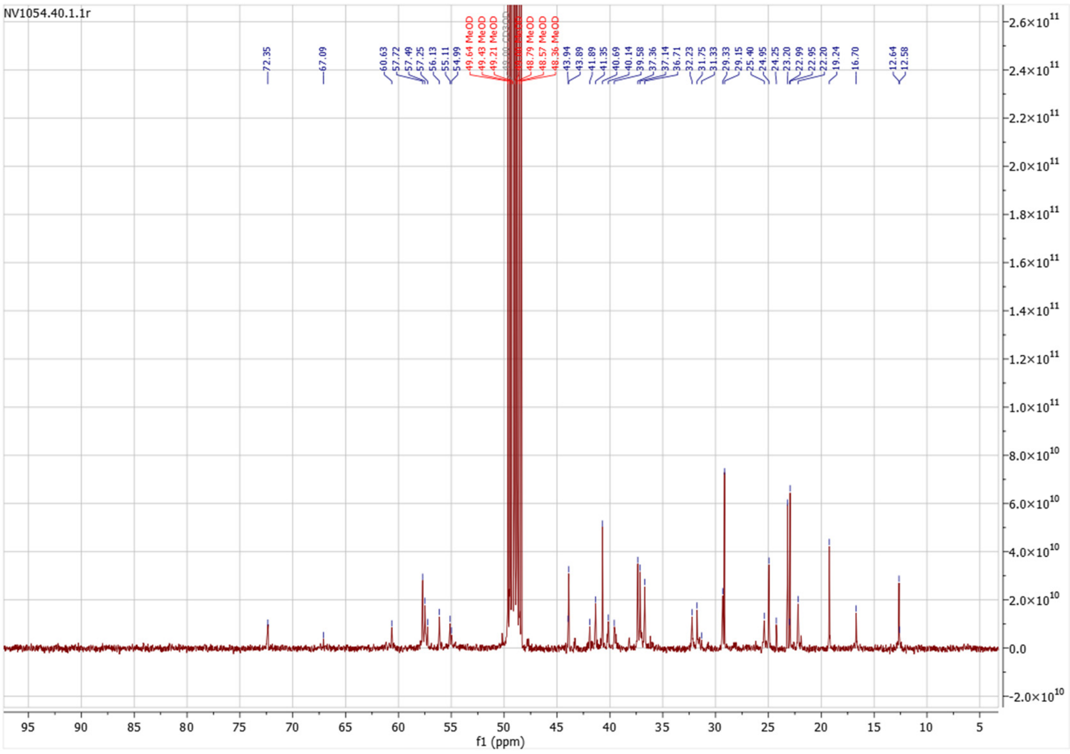

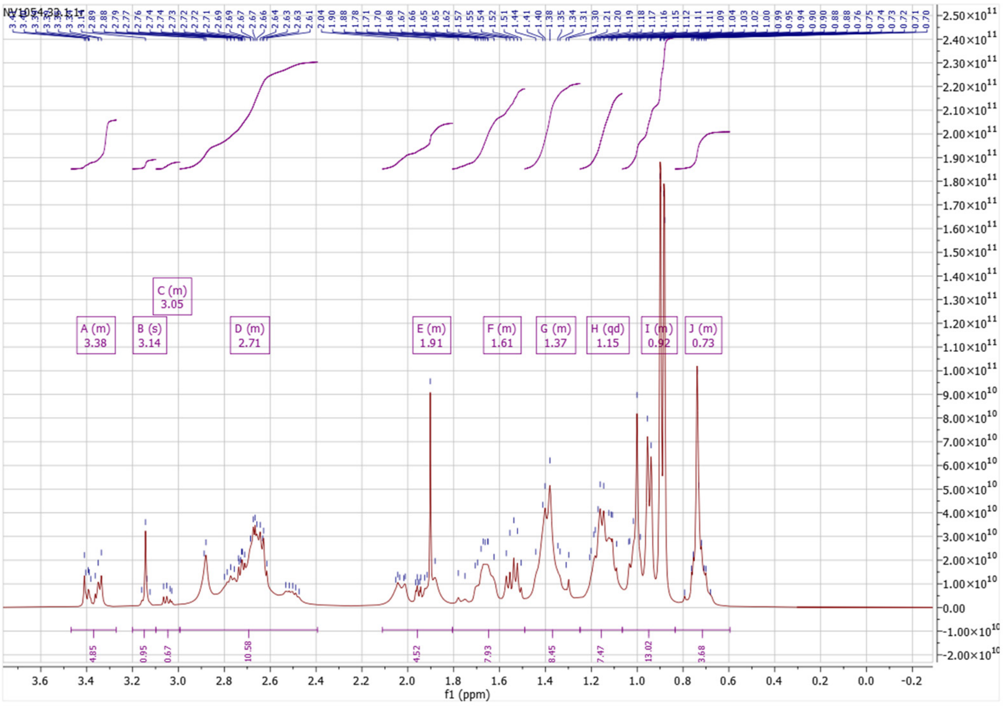

^{13}C and ^{1}H NMR spectra of 4m

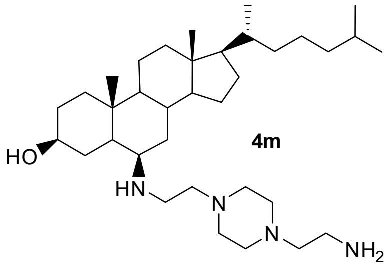

4m

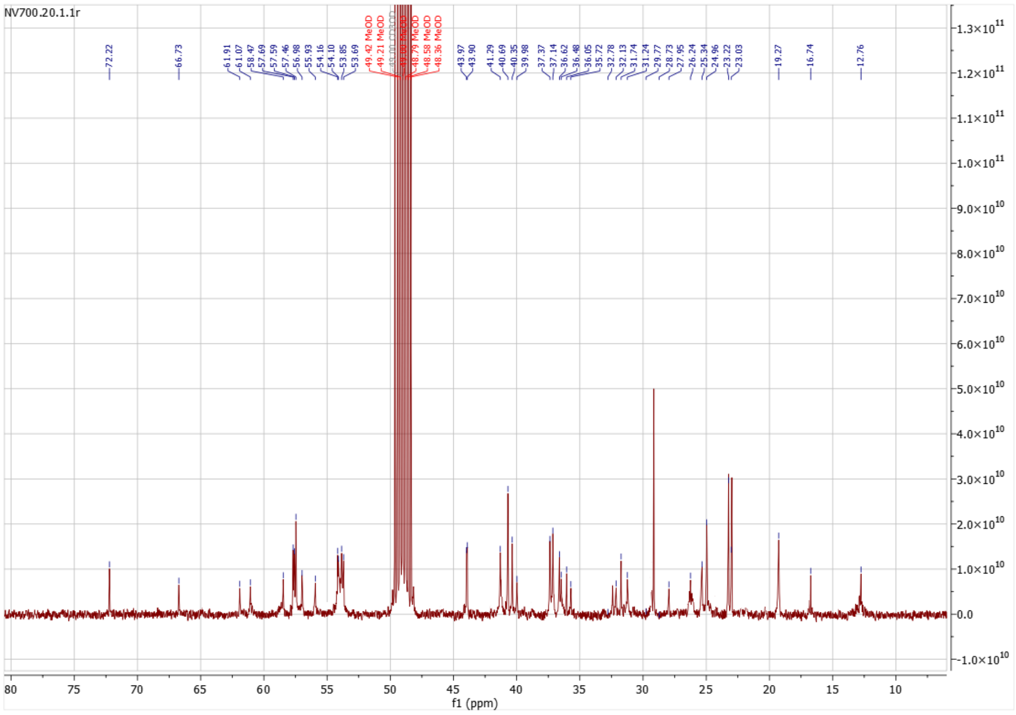

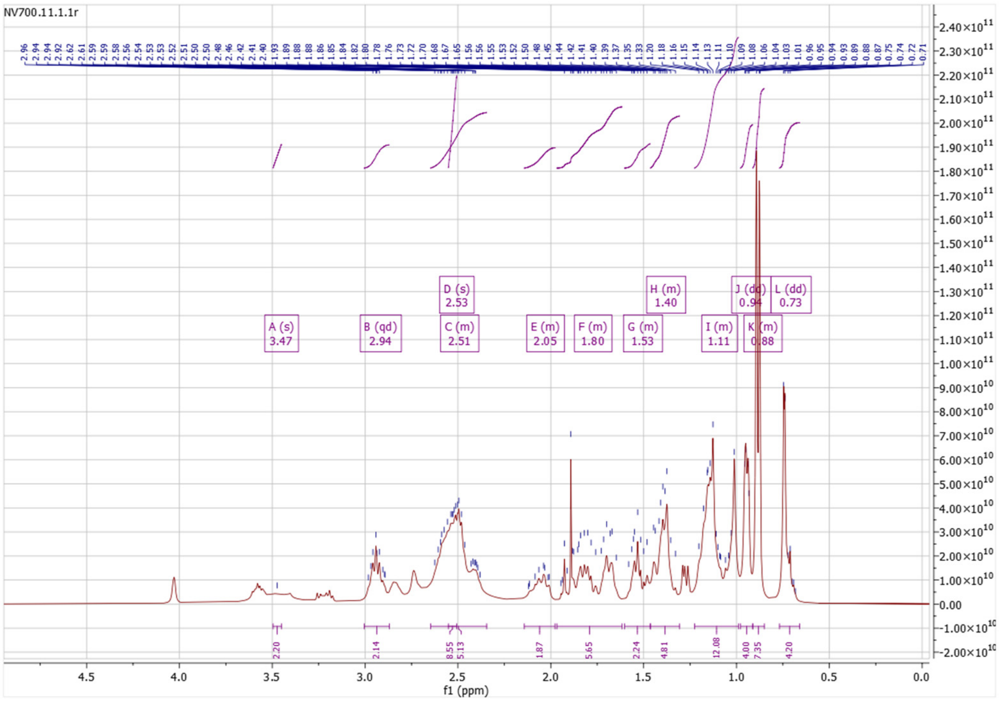

^{13}C and ^{1}H NMR spectra of 4n

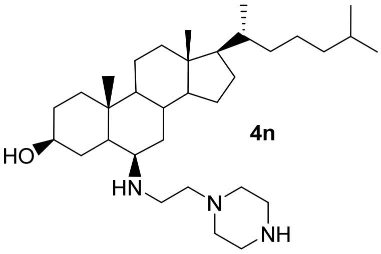

4n

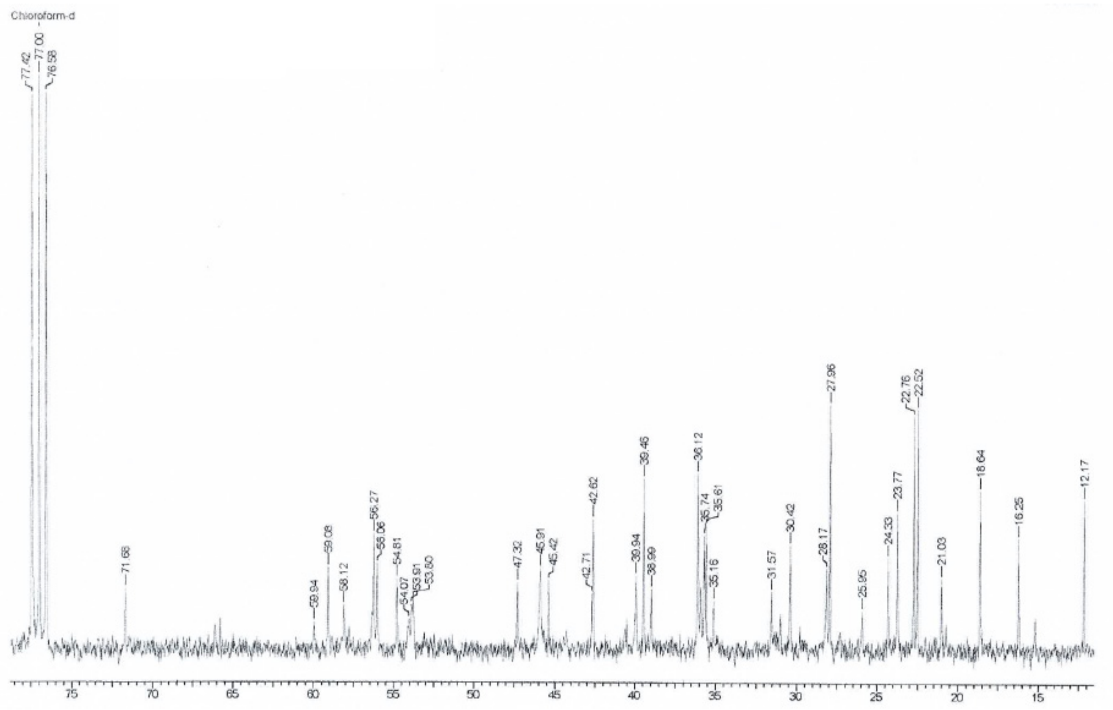

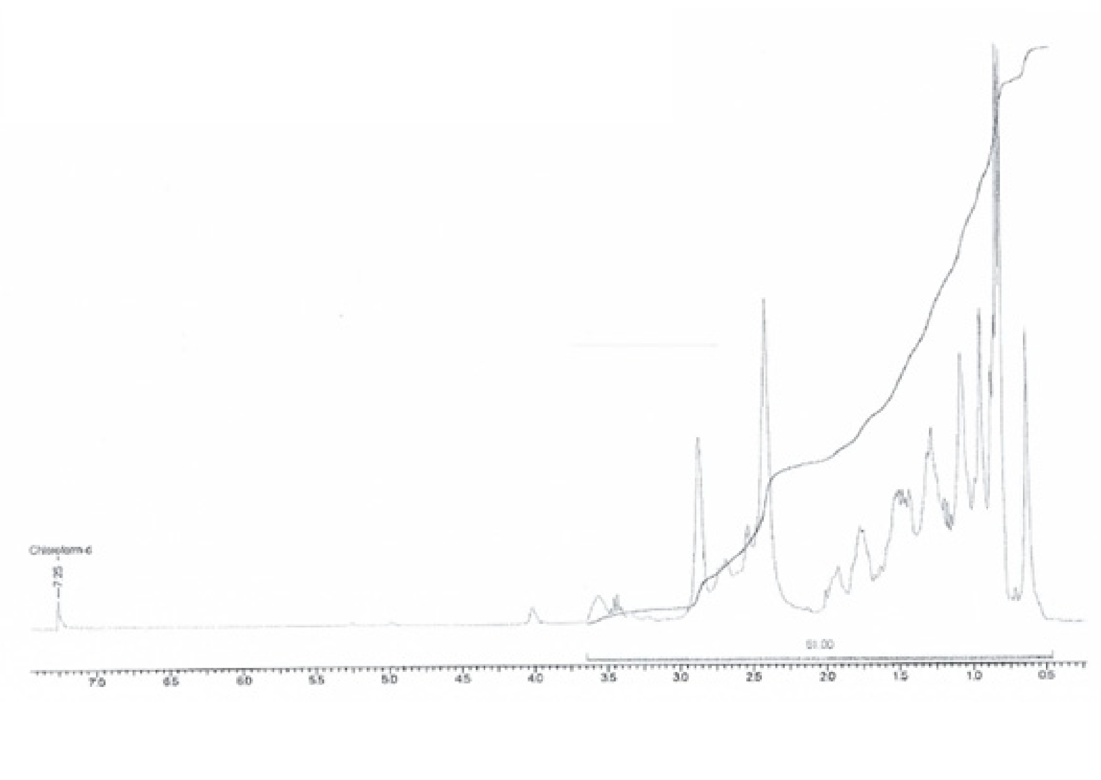

^{13}C and ^{1}H NMR spectra of 4o

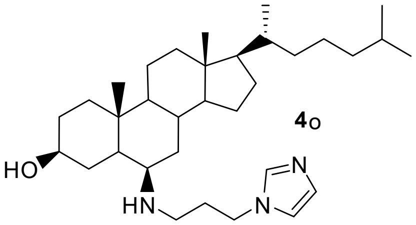

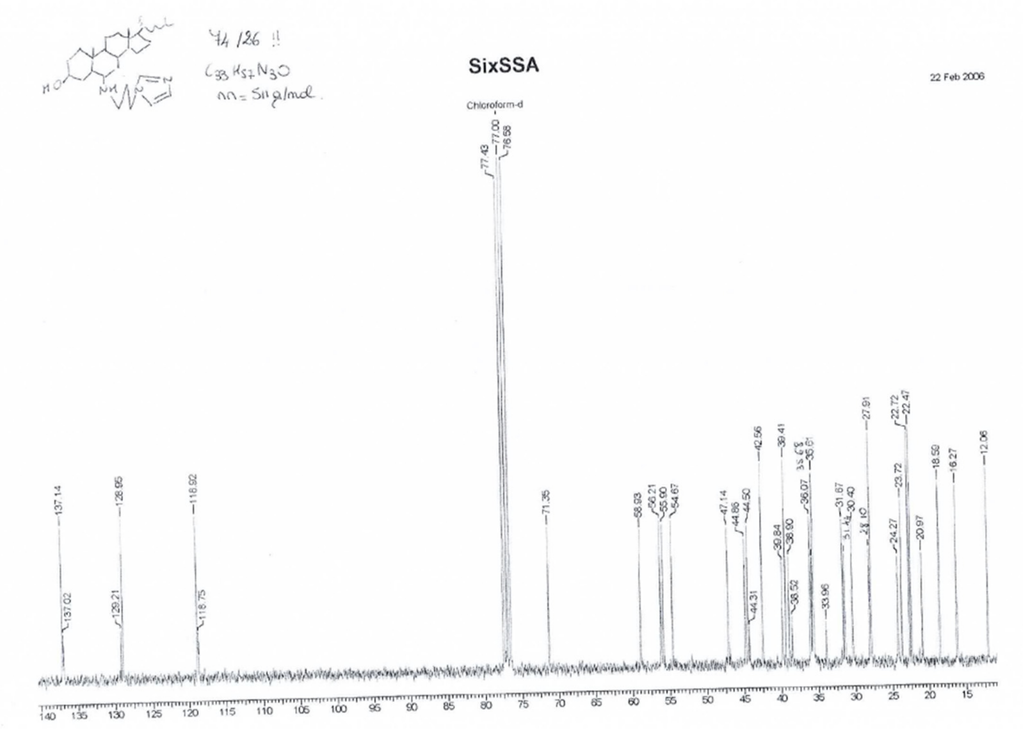

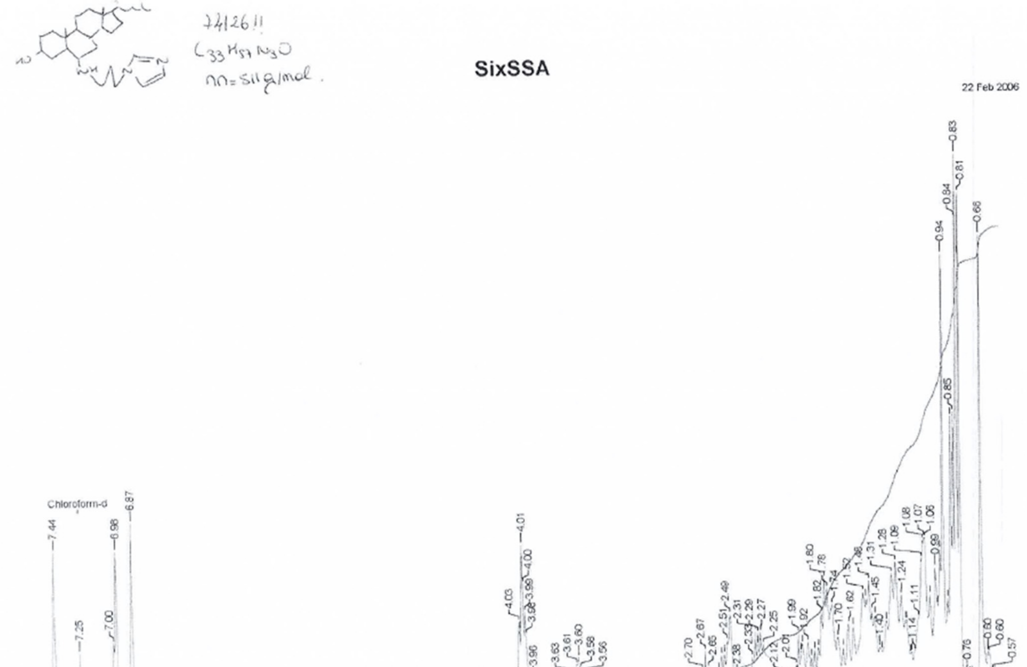

^{13}C and ^{1}H NMR spectra of 4p

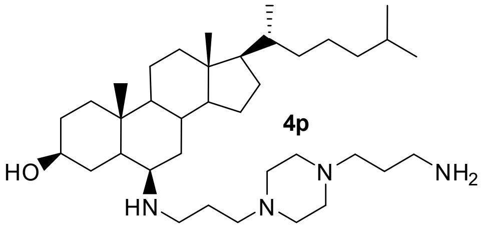

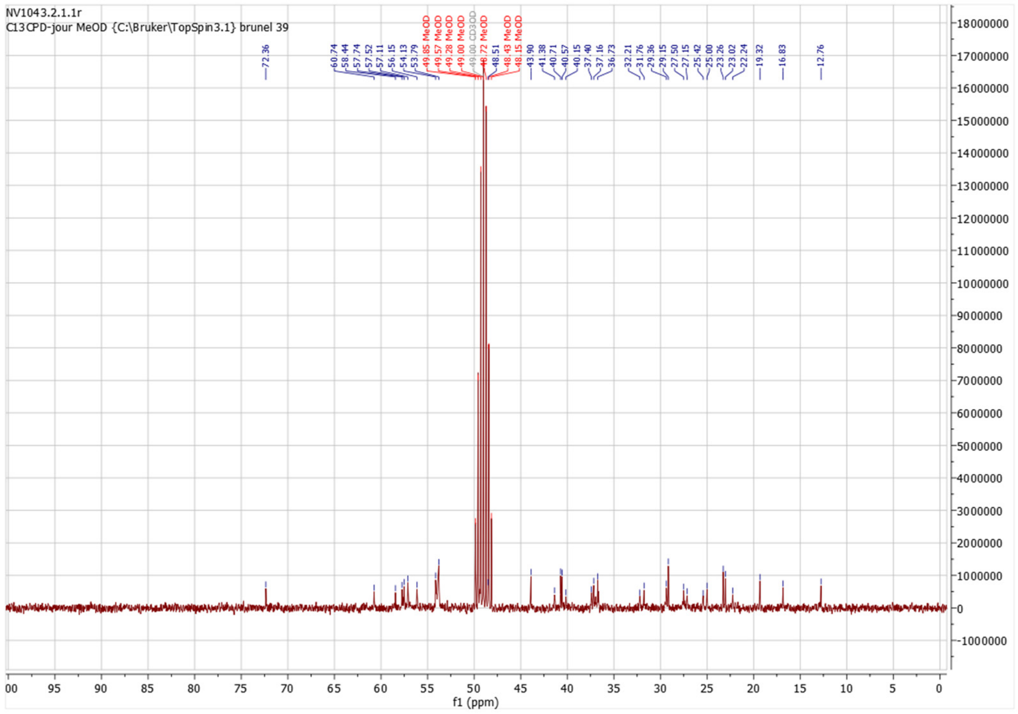

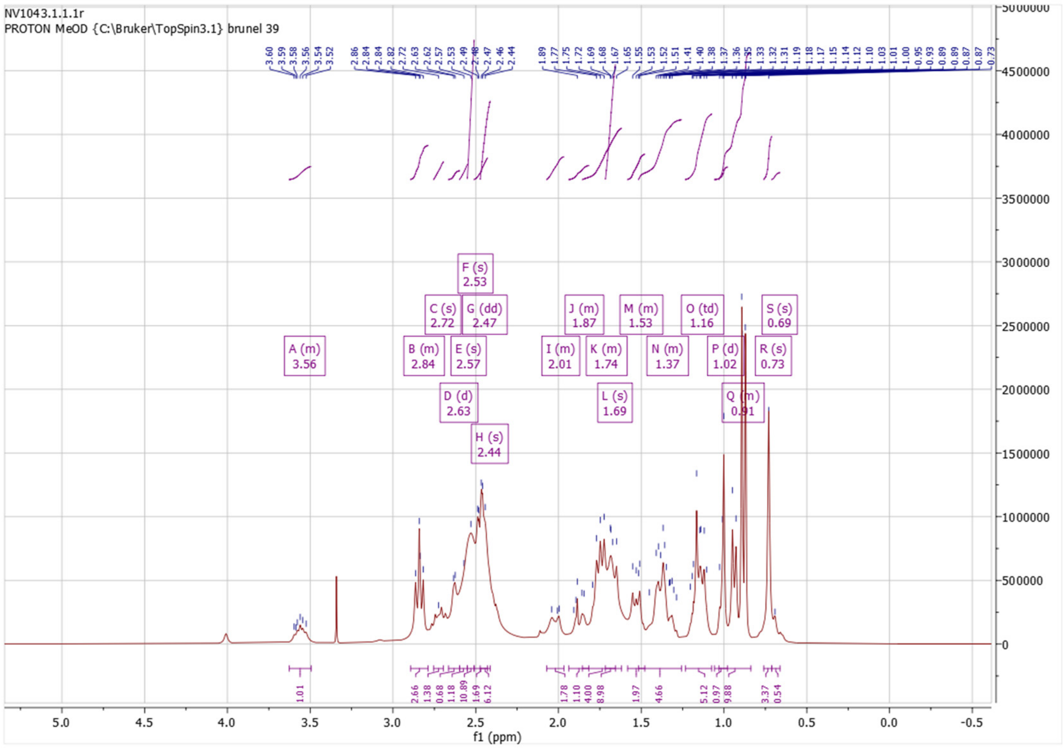

^{13}C and ^{1}H NMR spectra of 4q

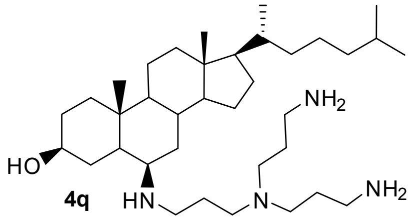

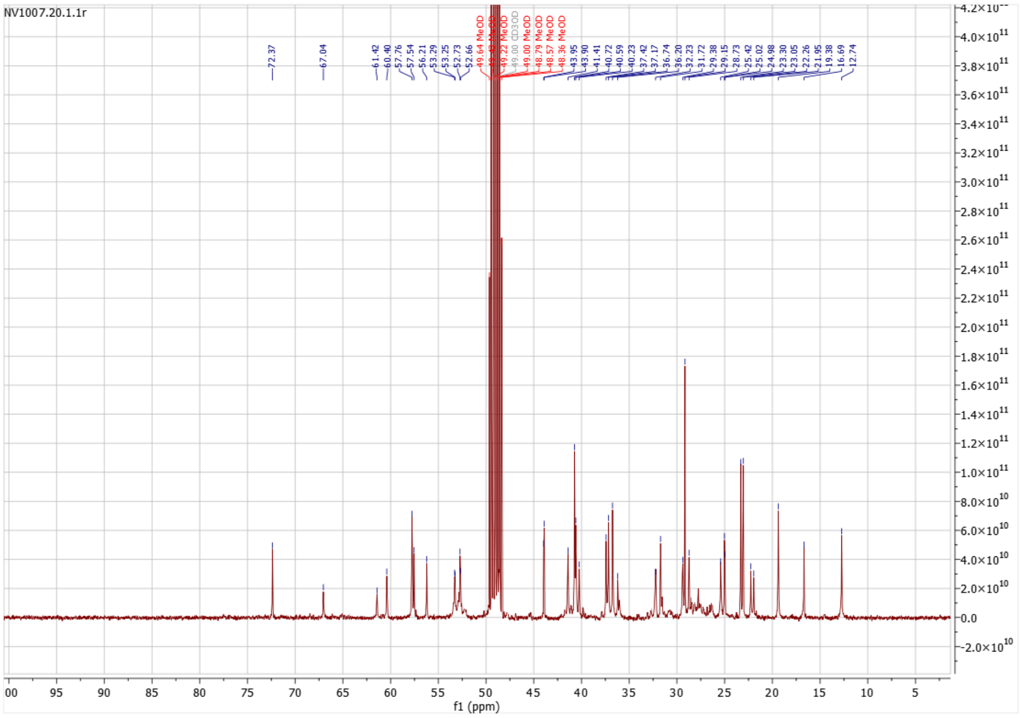

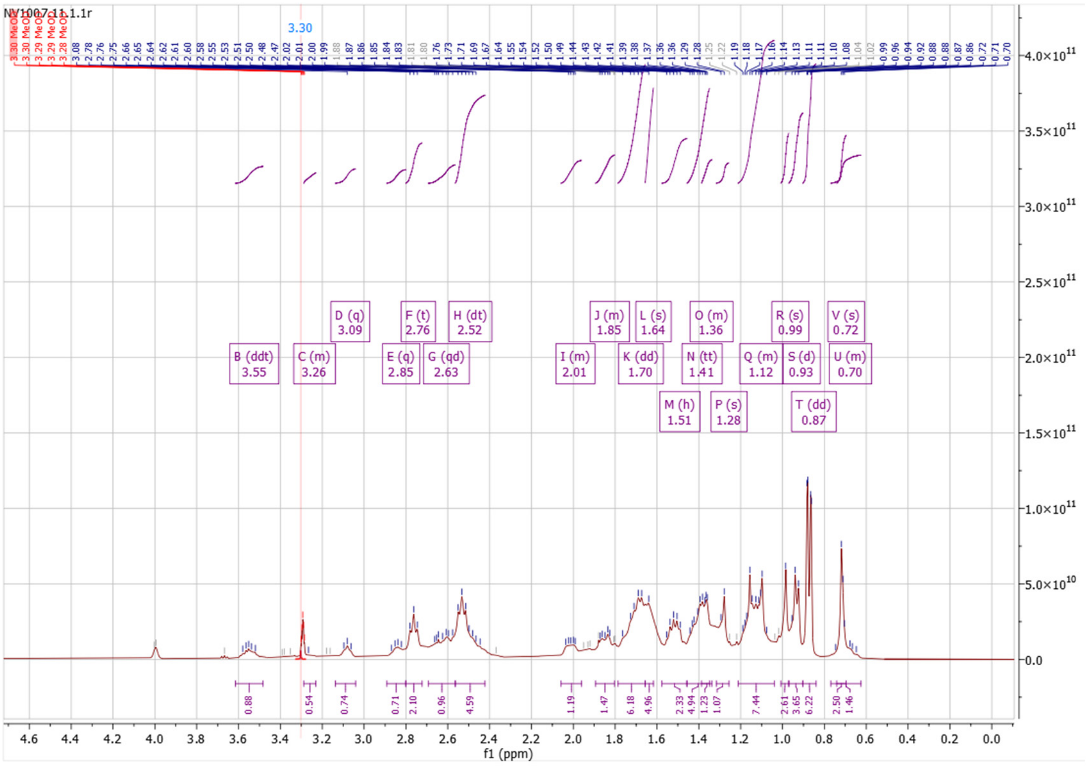

^{13}C and ^{1}H NMR spectra of 4r

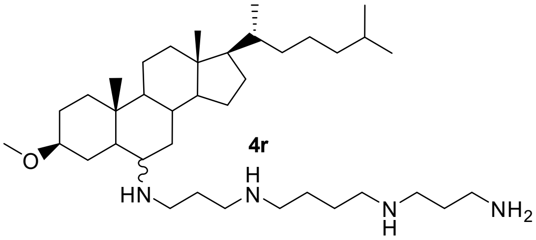

4r

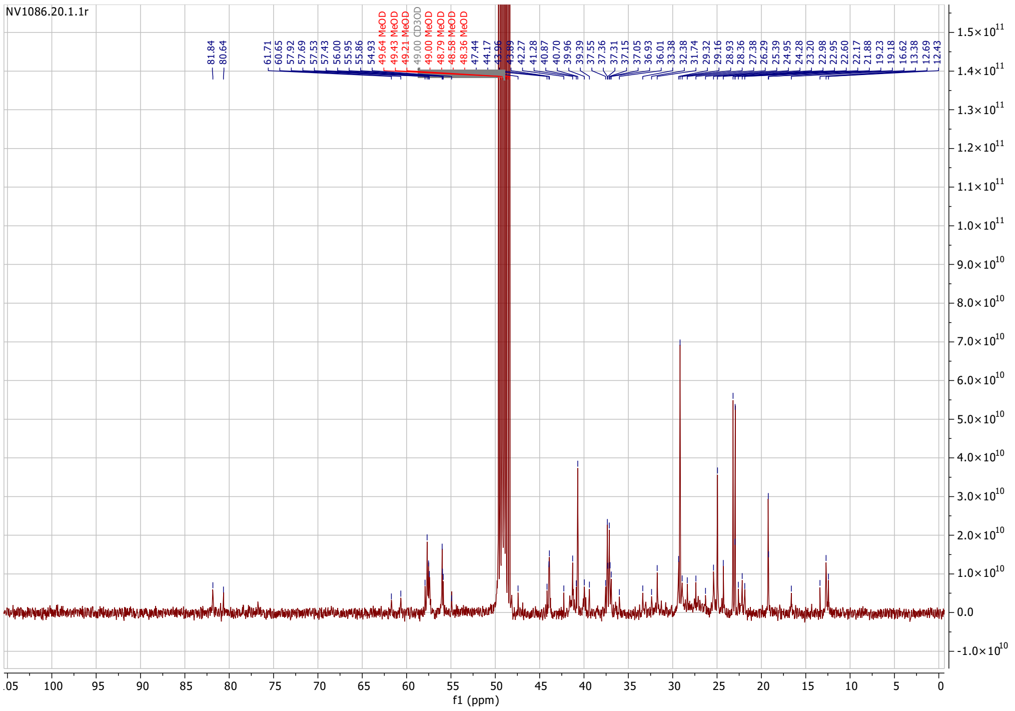

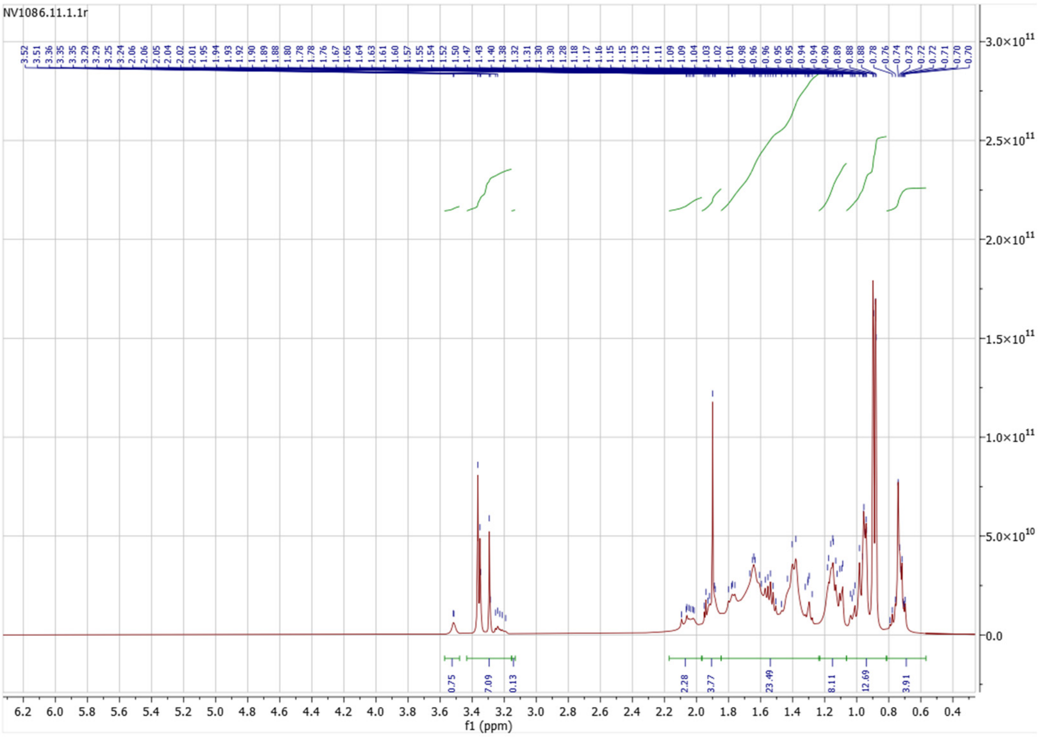

Supplement: Supplementary file 1 [file ijms-24-08568-s001.zip › ijms-2346120-supplementary.pdf]
